# Generative preparation tasks in digital collaborative learning: actor and partner effects of constructive preparation activities on deep comprehension

**DOI:** 10.3389/fpsyg.2024.1335682

**Published:** 2024-06-19

**Authors:** Stephan Mende, Antje Proske, Susanne Narciss

**Affiliations:** Psychology of Learning and Instruction, Technische Universität Dresden, Dresden, Germany

**Keywords:** digital collaborative learning, prior knowledge, text comprehension, learning activities, knowledge acquisition

## Abstract

Deep learning from collaboration occurs if the learner enacts interactive activities in the sense of leveraging the knowledge externalized by co-learners as resource for own inferencing processes and if these interactive activities in turn promote the learner's deep comprehension outcomes. This experimental study investigates whether inducing dyad members to enact constructive preparation activities can promote deep learning from subsequent collaboration while examining prior knowledge as moderator. In a digital collaborative learning environment, 122 non-expert university students assigned to 61 dyads studied a text about the human circulatory system and then prepared individually for collaboration according to their experimental conditions: the preparation tasks varied across dyads with respect to their generativity, that is, the degree to which they required the learners to enact constructive activities (note-taking, compare-contrast, or explanation). After externalizing their answer to the task, learners in all conditions inspected their partner's externalization and then jointly discussed their text understanding via chat. Results showed that more rather than less generative tasks fostered constructive preparation but not interactive collaboration activities or deep comprehension outcomes. Moderated mediation analyses considering actor and partner effects indicated the indirect effects of constructive preparation activities on deep comprehension outcomes via interactive activities to depend on prior knowledge: when own prior knowledge was relatively low, self-performed but not partner-performed constructive preparation activities were beneficial. When own prior knowledge was relatively high, partner-performed constructive preparation activities were conducive while one's own were ineffective or even detrimental. Given these differential effects, suggestions are made for optimizing the instructional design around generative preparation tasks to streamline the effectiveness of constructive preparation activities for deep learning from digital collaboration.

## 1 Introduction

This study aims to investigate whether having learners generate inferences from instructional material first on their own (i.e., enacting constructive activities) can prepare them for subsequently exploiting the potential benefits of digital collaboration in terms of using their co-learner's externalized knowledge as additional resource for own inferencing processes (i.e., interactive activities) in the service of in-depth knowledge acquisition (i.e., deep comprehension outcomes). In addition, the role of prior knowledge was taken into account.

To this end, we conducted a computer-supported collaborative learning (CSCL) experiment applying the so-called READ-script (Mende et al., 2017) where the members of learning dyads

a) read a text (reading phase),
b) prepared individually according to a certain preparation task (externalization phase),
c) exchanged each other's externalized task answers to inspect them (cognitive group awareness phase), and finally
d) entered a collaborative learning phase (discussion phase)

before answering a posttest capturing their deep text comprehension 1 week later. The type of the preparation task in the individual externalization phase was manipulated between experimental conditions in terms of the task generativity, that is, the extent of constructive preparation activities necessary to answer. Cognitive group awareness support was introduced in all conditions to facilitate dyad partner's immediate use of each other's preparation results for collaborative discussion, a strategy that has proven successful in research on individual preparation for collaborative learning (Mende et al., 2021).

**Figure 1:**
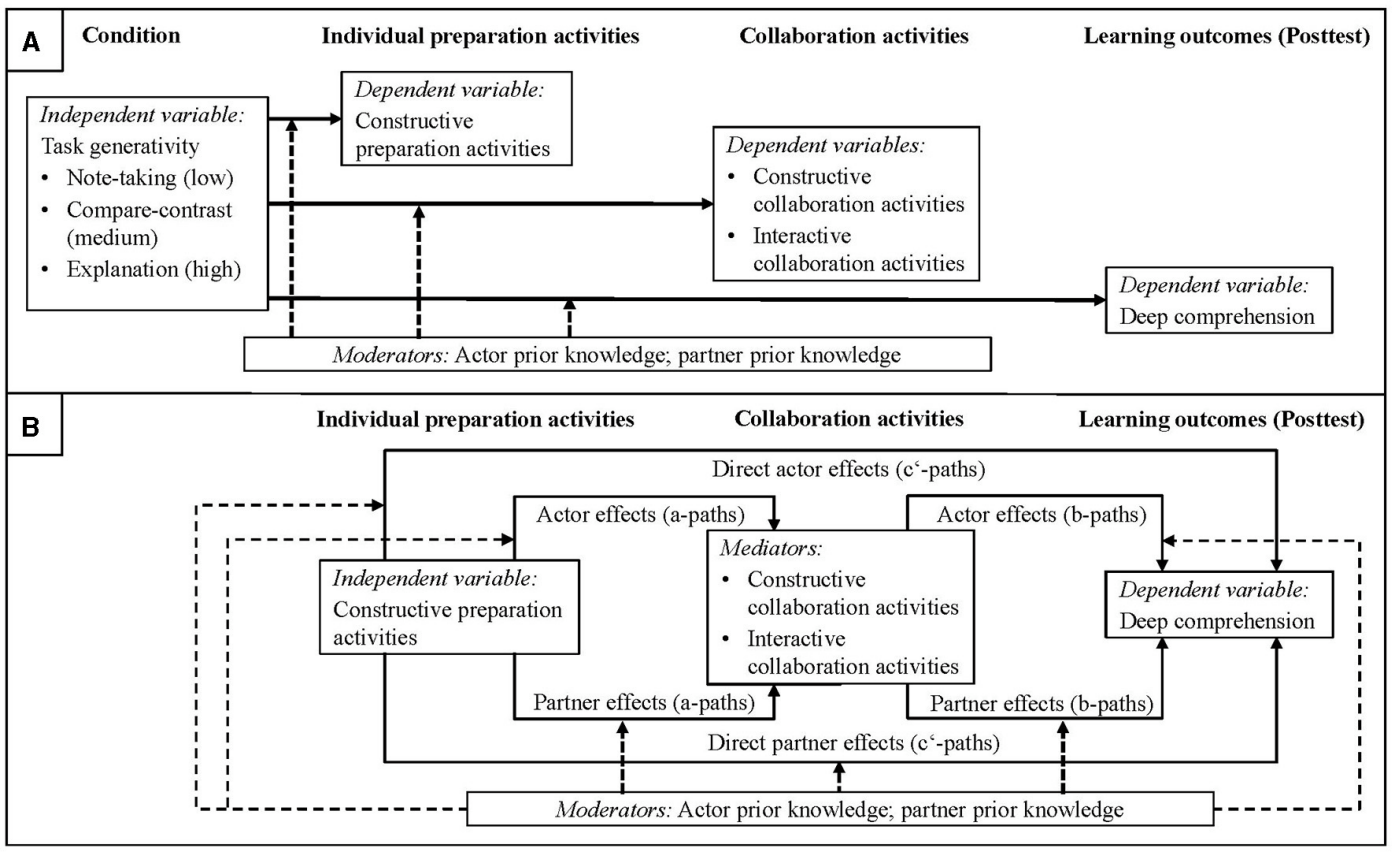
Summary of the relationships examined. **(A)** refers to research question 1. **(B)** refers to research question 2.

We firstly ask, on a more general level, whether more rather than less generative tasks intended to induce constructive preparation activities are suited to increase the execution of interactive activities and deep comprehension achievement while considering prior knowledge as potential moderator (research question 1; Figure 1A). We secondly ask on a more detailed level for the indirect and direct effects of the actor's and partner's constructive preparation activities on deep comprehension outcomes while considering constructive and interactive activities enacted during collaboration as potential mediators and prior knowledge as potential moderator (research question 2; Figure 1B).

### 1.1 Research background and motivation

Collaborative learning yields great potential, especially for university education (e.g., Scager et al., 2016). Besides helping to prepare students for professional life, in which teamwork plays a key role (e.g., De Hei et al., 2015) this is mainly because collaborative learning offers individual learners with enhanced opportunities to develop a deep comprehension of the instructional material in terms of well-connected and flexibly applicable knowledge (Fischer et al., 2013; Chi et al., 2018). However, it is often a challenge for practitioners to design collaborative learning scenarios in such a way that learners actually take advantage of these opportunities (Kirschner et al., 2009; Andrews and Rapp, 2015; Nokes-Malach et al., 2015; Jeong and Hmelo-Silver, 2016). Further, university students often perform their group work outside of the classes and, thus, away from the direct influence of their lecturers (Scager et al., 2016).

In this regard the use of digital technologies is promising, since it promotes better knowledge acquisition, more positive student perceptions and more effective group work and interaction compared to analog collaborative learning (Bromme et al., 2005; Chen et al., 2018). This is partly because (a) digital technologies can provide tools that enable more effective communication and facilitate the sharing of ideas, (b) learners have more time to think about and reflect on what other learners have contributed before responding, and (c) shy or passive learners also participate more in the interaction due to reduced psychological barriers, which promotes more equal communication and deeper discussions (Chen et al., 2018, p. 829). However, designing such digital technologies for collaborative learning is rather challenging (Narciss and Koerndle, 2008). Accordingly, research on computer-supported collaborative learning (CSCL) environments reveals the need to better understand how and under what conditions digital collaboration can promote in-depth knowledge acquisition (Jeong et al., 2019; Lämsä et al., 2021).

Recent theoretical developments aim to assist practitioners and researchers in a systematic consideration of the factors and processes relevant to the success of (CS)CL. Input-process-outcome models (e.g., Dillenbourg et al., 2009; Deiglmayr et al., 2015; Janssen and Kirschner, 2020) emphasize that a given collaborative learning instruction (*input*) does not lead to deep comprehension (*outcome*) directly but mediated through the activities (*processes*) actually executed by the learners during collaboration. This points at the need to identify effective learning activities and to evaluate (a) whether the provided input actually induces learners to execute these activities during collaboration as well as (b) whether these activities actually promote deep comprehension outcomes. These input-process as well as process-outcome relationships may be moderated by further variables such as learner characteristics (e.g., Deiglmayr et al., 2015). Furthermore, information processing-oriented benefit-cost approaches (Nokes-Malach et al., 2015; Janssen and Kirschner, 2020; Mende et al., 2021) highlight that collaboration may not necessarily have only conducive but can also have hindering effects on the individual's learning. This should be considered as well to obtain a complete picture of why a collaborative learning instruction (does not) work and how to (further) optimize it.

In terms of processes, students can principally perform various learning activities during collaboration (e.g., Vogel et al., 2017). The ICAP framework (Chi, 2009) allows classifying these activities in order to derive predictions regarding their effects on learning. Thereby, so-called *constructive* and *interactive* activities are considered suitable to promote deep comprehension (Chi and Wylie, 2014). Both constructive and interactive activities involve the externalization of content-relevant information that is not originally given in the instructional material but is generated or inferred from it. Different to a constructive activity, an interactive activity is additionally characterized by taking into account or referring to a co-learner's externalized knowledge. That is, a learner is said to perform an interactive activity, when they refer to information contributed by a co-learner and, incorporating it, infer new information beyond what is already given. Hence, while constructive activities can be performed irrespective of whether learning alone or in a group, interactive activities presuppose a collaborative learning situation and are characterized herein by leveraging the knowledge of co-learners as a resource for own inferencing processes (Chi and Wylie, 2014; Chi et al., 2018).

Thus, adopting a benefit-cost perspective of the single learner working with other co-learners, the potential benefits of collaboration arise particularly from the co-learners constructive and/or interactive activities: they result in externalizations of new information not already contained in the presented instructional material which the learner could use as additional learning resource in the service of in-depth knowledge acquisition. Doing so requires the learner to actually execute interactive activities which can therefore be understood as the active process of using the potential benefits of collaboration (Fischer et al., 2013; Janssen and Kirschner, 2020; Chi, 2021).

At the same time, as already mentioned, collaboration yields not only potential benefits but also costs for the learners' information processing (e.g., Mende et al., 2021). These costs are associated with the presence as well as the use of externalizations from other co-learners: being exposed to the externalizations of co-learners yields the risk of interferences and being disrupted in one's own train of thought (e.g., Nijstad and Stroebe, 2006; Rajaram and Pereira-Pasarin, 2010; Nokes-Malach et al., 2015). Further, the dual task of processing not only the instructional material but also the information externalized beyond it by co-learners often challenges the individual learner's cognitive capacities (e.g., Dillenbourg and Betrancourt, 2006; Kolfschoten et al., 2014). Moreover, using co-learners' externalizations by executing interactive activities is associated with additional coordination and communication demands, further burdening the learners' information processing resources (Janssen et al., 2010; Janssen and Kirschner, 2020). This is even more crucial in university settings where learning partners are not permanently together in stable classes and, thus, not necessarily know each other before engaging in joint group work (Scager et al., 2016). Associated with this, university students often tend to focus primarily on the task and less on the team aspect of collaborative learning. However, the effectiveness of collaborative learning heavily depends on how the interaction between the learners as well as the individual accountability of the single learners for the group work is organized (Fransen et al., 2011; De Hei et al., 2015).

Therefore, collaborative learning does often not promote deep comprehension because learners cannot deal with the costs or doing so does prevail the potential benefits (Bromme et al., 2005; Nokes-Malach et al., 2015; Menekse and Chi, 2019; Janssen and Kirschner, 2020). This calls for support strategies that can help to raise the benefits and to reduce the costs, so that the execution of interactive activities is promoted and, thus, the collaborative learning potentials for in-depth knowledge acquisition can unfold.

In addressing these issues, a variety of support strategies have been developed in the last years. Digital technologies are even accelerating this trend, as they enable enhanced communication, increased productivity, flexibility, as well as scalability compared to analog solutions. This enables a more efficient and flexible implementation of more comprehensive forms of guidance, scaffolding and tools (Dillenbourg et al., 2009; Jeong and Hmelo-Silver, 2016). A frequently applied strategy consists in preceding collaborative learning with a phase in which learners first execute activities directed at processing the instructional material on their own such as writing down notes or explanations (i.e., individual preparation for collaborative learning; van Boxtel et al., 2000; Tsovaltzi et al., 2015; Lam and Kapur, 2017; Mende et al., 2021). This strategy is often complemented by a specific form of cognitive group awareness support (e.g., Janssen and Bodemer, 2013) in the sense of making the externalizations created during individual preparation (e.g., written notes or explanations) available to all learners in the group as a resource for the subsequent collaboration phase (e.g., Gijlers et al., 2013; Engelmann et al., 2014). The former targets at learners first activating their prior knowledge and building up an initial understanding of the instructional material without the additional demands of collaboration in order to have an expanded knowledge base and more free cognitive capacities to process and integrate co-learners' contributions in subsequent collaboration (Lam and Kapur, 2017; Tsovaltzi et al., 2017; Mende et al., 2021). The latter aims at providing learners with information about the knowledge, perspectives and ideas of their co-learners so that their individually externalized information can be accessed directly in collaboration and further the mutual communication and coordination is facilitated (e.g., Janssen and Bodemer, 2013; Noroozi et al., 2013; Erkens et al., 2016; Jeong and Hmelo-Silver, 2016). Hence, both strategies are intended to improve the benefit-cost ratio of executing interactive activities.

While research indicates the combined use of individual preparation and cognitive group awareness support to be suited to promote interactive activities during and deep comprehension outcomes from collaborative learning (Mende et al., 2021), an open question remains as to what kind of preparation activities learners should enact in such a setting. In principle, learners could execute a variety of activities that might be differently productive for (subsequent) learning. For example, they could restate what is already presented in the instructional material, an activity typically considered to correspond with more shallow information processing (e.g., King, 1999; Roscoe, 2014; Chi et al., 2018). In contrast, some recent work has argued that learners should engage in deeper information processing already during individual preparation by going beyond the given instructional material through the execution of constructive activities. Executing these constructive preparation activities is hypothesized to help learners exploiting the potential benefits of subsequent collaboration in terms of deep comprehension outcomes (Lam and Kapur, 2017; Lam and Muldner, 2017; cf. Mende et al., 2021). While these assumptions are well grounded in previous theoretical and empirical work concerned with individual learning (e.g., Wittrock, 1989; Schwartz et al., 2011; Kapur, 2015), they rarely have been subjected to an empirical investigation so far in CSCL research.

### 1.2 Research approach and objectives

The present study aims at contributing to extend the existing research by adopting a benefit-cost perspective considering input, processes, and outcomes. On the one hand, it is of interest how to induce learners to execute constructive activities during individual preparation as a prerequisite for the proposed beneficial effects on subsequent collaborative learning processes and outcomes coming into effect. In this regard some previous research has addressed the role of the preparation task *generativity*, that is, its potential to induce constructive activities (e.g., Lam and Kapur, 2017; Lam and Muldner, 2017). In addition, research has suggested that learners' capabilities to perform constructive activities is strongly affected by their prior knowledge (e.g., Kintsch, 2004; Best et al., 2005; McNamara and Magliano, 2009). Accordingly, the latter may moderate respective task effects.

On the other hand, it is of interest whether the execution of constructive activities during individual preparation indeed promotes the learner's personal exploitation of the potential collaboration benefits for in-depth knowledge acquisition. In order to obtain a comprehensive and informative picture we argue that an appropriate investigation of this question requires the consideration of the following three points: first, one's own and one's collaboration partner's enacted learning activities do not necessarily relate in the same way to one's personal collaboration benefits and costs (e.g., Vogel et al., 2016). Accordingly, it is necessary to examine the effects of each learners' preparation and collaboration activities both on their own and each other's subsequent learning processes and/or outcomes. Second, self-performed interactive activities are considered the personal process of actively using the potential benefits of collaboration for in-depth knowledge acquisition. Consequently, addressing the question of whether deep learning *from* collaboration can be fostered by one's own and/or one's co-learner's previously executed constructive *preparation* activities requires examining the latter two in view of their indirect effects on one's own deep comprehension outcomes that are mediated trough these self-performed interactive collaboration activities. Third, previous research suggests, among others, the learners' prior knowledge to be a crucial impact factor for the personal benefit-cost-ratio of collaborative learning (e.g., Nokes-Malach et al., 2012, 2015; Kirschner et al., 2018; Janssen and Kirschner, 2020). Consequently, prior knowledge should be taken into account as a potential moderator regarding the outlined relationships.

In order to comply with the analytical requirements described, we conducted moderated mediation analyses (e.g., Hayes, 2013) accounting for the distinct contributions of the learner's own as well as their co-learner's preparation and collaboration activities. For the case of dyads (i.e. groups of two) this differential consideration can be taken into account with the actor-partner interdependence model (Kenny et al., 2006). Within this analytic approach dyadic influences are differentiated in terms of actor and partner effects. Actor effects, on one hand, refer to *intrapersonal* relationships between variables within the same person, for example, the effect of self-performed constructive preparation activities on subsequently self-performed interactive collaboration activities. Partner effects, on the other hand, refer to *interpersonal* relationships between variables of different persons, for example, the effect of co-learner's constructive preparation activities on subsequently self-performed interactive collaboration activities or the effect of self-performed constructive preparation activities on co-learner's subsequent interactive collaboration activities, respectively (Kenny et al., 2006).

Before we showcase the present study we first discuss the effects of the preparation task generativity on the execution of constructive activities. Afterwards we address the complex dynamics that may underlie the effects of constructive preparation activities on deep learning from subsequent collaboration considering an actor and a partner perspective. This is followed by addressing the potentially moderating role of prior knowledge.

### 1.3 The effects of task generativity on constructive preparation activities

Constructive preparation activities can unfold their potential advantages for subsequent collaboration processes and outcomes only, if learners indeed enact them (cf. Chi and Wylie, 2014). Yet, learners often tend to restate information already given in the instructional material instead of drawing inferences going beyond, even when they are asked to do the latter (e.g., Chi et al., 2018; Chase et al., 2019). This raises the question of how and under what conditions learners are executing constructive preparation activities. One important variable in this regard is preparation task type (e.g., Lam and Kapur, 2017; Lam and Muldner, 2017).

Preparation tasks can differ in their potential to induce constructive activities. Inspired by Lam and Kapur (2017) we use the term preparation task *generativity* to this end, which could be defined as the extent to which the task requires the learner to infer and externalize information beyond the given instructional material by connecting the to-be-learned information with each other and/or with their pre-existing knowledge. In other words, the higher the task generativity, the more constructive activities are necessary to answer (e.g., Grabowski, 2004; Chin et al., 2016; Fiorella and Mayer, 2016; Brod, 2020; Morris and Chi, 2020).

Generative learning research has addressed various tasks differing in their generativity. One often considered task is note-taking (e.g., Grabowski, 2004; Stefanou et al., 2008; Fiorella and Mayer, 2016). Unless provided with further specifications, note-taking tasks do not explicitly require learners to go beyond what is already given in the instructional material. Accordingly, there is a huge variability concerning what learners actually do in response to such tasks. Though learners could, in principle, add new content when taking notes, for instance, in the form of inserting unstated links between the received information or writing down own examples for to-be-learned concepts or principles. However, learners often seem more prone to simply restate the information explicitly given in the instructional material (e.g., Grabowski, 2004; Igo et al., 2008; Miyatsu et al., 2018; Ponce et al., 2020).

Two other generative tasks commonly applied in individual learning research concerned with preparing students for learning target content from subsequent lectures are compare-contrast tasks and explanation tasks (cf. Roelle and Berthold, 2016). The former prompt learners to find similarities and differences between contrasting cases, concepts or the like (e.g., Schwartz and Bransford, 1998). The latter go beyond this by asking learners to generate an explanation for the similarities and differences (e.g., Schwartz and Martin, 2004). Thus, while both tasks require the execution of constructive activities to answer, explanation tasks do so to a higher extent than compare-contrast tasks since more inferences are required. This consideration is in line with research suggesting that explaining the relations between contrasting cases prepares better for subsequent deep learning than simply comparing contrasting cases (Sidney et al., 2015; Chin et al., 2016). Taken together, the three tasks could be arranged according to their relative generativity in increasing order from note-taking (low) to compare-contrast (moderate) to explanation (high).

### 1.4 The actor and partner effects of constructive preparation activities on post-collaborative deep comprehension outcomes: toward a moderated mediation model

Does the execution of constructive preparation activities indeed promote the individual learner's personal exploitation of the potential benefits of subsequent collaboration for in-depth knowledge acquisition? As outlined, we argue that this could only be said if, in the sense of indirect effects (formally called a^*^b-paths), the actors and/or the partner's constructive preparation activities actually foster the actor's interactive collaboration activities (a-paths) and the latter in turn indeed promote the actor's deep comprehension outcomes (b-path, Figure 1B). Investigating this question requires mediation analyses that examine the occurrence of such indirect effects while simultaneously controlling for the direct effects (c'-paths), that is, the effects of constructive preparation activities on deep comprehension outcomes that are not transmitted by the potential mediators under consideration (e.g., Zhao et al., 2010). Hence, in the following sections we elaborate on the potential actor and partner effects of (a) constructive preparation activities on interactive collaboration activities (a-paths) and (b) of interactive collaboration activities on deep comprehension outcomes (b-paths).

#### 1.4.1 Actor and partner effects of constructive preparation activities on interactive collaboration activities (a-paths)

As described, a learner's execution of interactive activities during collaboration may depend on whether the associated coordination and communication costs can be dealt with and whether doing so pays off (e.g., Janssen and Kirschner, 2020). Especially when individual preparation is complemented by group awareness support, this personal benefit-cost-ratio may be affected not only by one's own preceding constructive preparation activities (actor effect) but also by the constructive preparation activities performed of one's co-learner (partner effect). In this regard, both the actor's self-performed and the partner's enacted constructive preparation activities may each not only yield potential advantages but also disadvantages:

Adopting an *actor perspective*, research shows that the execution of constructive activities fosters deep comprehension outcomes (e.g., McNamara and Magliano, 2009; Ozuru et al., 2010; Chi and Wylie, 2014; Roscoe, 2014; Roelle et al., 2015). Therefore, the number of constructive activities the actor executes when studying the instructional material during an individual preparation can be expected to foster the coherence and comprehensiveness of his or her initial understanding of the to be learned information prior to collaboration. Hence, in view of the subsequent collaboration, constructive preparation activities may positively affect (a) the learner's initial knowledge base upon which the additional information externalized by the co-learner could be integrated in terms of interactive activities and (b) the cognitive capacities available to deal with the associated coordination and communication costs (Schwartz et al., 2007; cf. Lam and Kapur, 2017; Tsovaltzi et al., 2017; Mende et al., 2021; Tan et al., 2021). Since in the same breath, however, the gaps between the actor's knowledge and the to-be-learned instructional material should be reduced through constructive preparation activities, the latter may also decrease the (experienced) potential benefits of subsequently using the co-learner's externalizations as additional learning resource (cf. Janssen and Kirschner, 2020). Therefore, it could also be that constructive preparation activities reduce the execution of interactive activities, possibly in favor of more individualistic learning processes (e.g., Tsovaltzi et al., 2015, 2017) such as the continued execution of constructive activities during collaboration which are not associated with communication and coordination costs and, thus, might yield a better benefit-cost ratio.

Considering the *partner perspective*, while own constructive activities correspond to own in-depth knowledge acquisition processes, the co-learner's constructive activities *per se* only represent additional information to oneself at first (e.g., Vogel et al., 2016). More concretely, the more constructive preparation activities are carried out by the partner, the more additional ideas, knowledge, and conclusions are externalized and presented to the actor in the course of group awareness support. Thus, on the one hand, the more constructive preparation activities executed by the partner, the more information not contained in the previously studied material are available to the actor right at the start of collaboration. Hence, the partner's constructive preparation activities increase the potential collaboration benefits for in-depth knowledge acquisition which the actor could use by performing interactive activities (Chi and Wylie, 2014; Chi et al., 2018). For example, the additional information provided by the partner can aid the actor in activating task relevant knowledge (cross cueing; e.g., Wegner, 1987; Moreland and Myaskovsky, 2000; Marion and Thorley, 2016) and induce conceptual cognitive conflicts (e.g., King, 1999; Cress and Kimmerle, 2008; Jorczak, 2011; Slavin, 2011; Webb, 2013) which in turn may assist, stimulate or provoke the actor to draw (further) inferences conducive to his or her own deep comprehension (cf. Dugosh et al., 2000; Noroozi et al., 2013; Erkens et al., 2016). On the other hand, however, this additional information also increases the overall complexity of the learning environment, putting additional burdens on the actor's cognitive resources (Dillenbourg and Betrancourt, 2006; Kolfschoten et al., 2014). In other words, the partners' constructive preparation activities increase the information processing costs the actor has to deal with and therefore may impede his or her execution of activities that correspond to deep information processing, such as interactive activities (Kirschner et al., 2018; Janssen and Kirschner, 2020; Mende et al., 2021).

#### 1.4.2 Actor and partner effects of interactive collaboration activities on deep comprehension outcomes (b-paths)

As is the case with constructive activities, also self-performed and co-learner enacted interactive activities can be considered to relate differently to the individual learner's personal costs and benefits that are associated with collaboration (e.g., Chi and Wylie, 2014). Similar to self-performed constructive activities, also the actors own interactive activities can be expected to foster deep comprehension outcomes (King, 1999; Chi, 2009; Chi and Wylie, 2014; Deiglmayr and Schalk, 2015; Mende et al., 2017). Compared to constructive activities, only these interactive activities actively use the potential benefits of collaboration to this end (e.g., Chi et al., 2018).

In contrast, the results of the partner's interactive collaboration activities *per se* only provide additional information to the actor that is not contained in the instructional material—similar to the partner's constructive preparation or collaboration activities. Such additional information is important but not sufficient for the actor to benefit from collaboration in terms of deep comprehension outcomes. To this end, the externalizations resulting from the partners constructive or interactive activities must be subjected to the actor's interactive activities (Chi and Wylie, 2014; Chi et al., 2018). This view is supported by previous research suggesting that just receiving explanations from others does often not foster learning unless the explanations are elaborated or further applied by the receiver (Webb and Mastergeorge, 2003; Wittwer and Renkl, 2008; Vogel et al., 2016). Thus, when considered simultaneously, the actors but not necessarily the partner's interactive activities could be expected to foster the actor's deep comprehension outcomes. Moreover, since the partners interactive (and constructive) activities do not only represent additional resources (i.e., potential benefits) but at the same time increase the information processing demands (i.e., costs) for the actor, even the possibility of negative partner effects must be taken into account (cf. Dillenbourg and Betrancourt, 2006; Nokes-Malach et al., 2015; Janssen and Kirschner, 2020).

### 1.5 The potentially moderating role of prior knowledge

Prior knowledge could be understood as the amount of information related to the target instructional material already stored in a learner's long-term memory at the start of a learning phase (e.g., McCarthy and McNamara, 2021; Simonsmeier et al., 2022). Generally, learners already possessing high topic relevant prior knowledge are better able to activate relevant knowledge structures from their long-term memory in order to relate them to incoming information while low prior knowledge learners are less so. Accordingly, prior knowledge guides processing of novel information and fosters the construction and integration of knowledge from that information (e.g., Kintsch, 1998, 2004; Best et al., 2005; Kalyuga, 2009; McNamara and Magliano, 2009; Witherby and Carpenter, 2021). Hence, prior knowledge represents a crucial factor determining learners' capabilities to perform learning activities involving inferences, such as constructive or interactive activities (e.g., Webb, 1989; Chan et al., 1992; Kintsch, 1998; Ertl et al., 2004; McNamara, 2004; Best et al., 2005; Schwartz et al., 2007; Chi and Wylie, 2014). Consequently, prior knowledge could play a role for the effects investigated in this study in several respects.

Firstly, prior knowledge may play a role for whether and in which quantity learners indeed execute the constructive activities they are asked for by a generative preparation task. Prior research suggests that generative instructions and tasks are more effective for high than for low prior knowledge learners in terms of knowledge acquisition (Kirschner et al., 2006; Chen et al., 2017). Consequently, the effects of individual preparation task generativity on the execution of constructive preparation activities may increase with increasing prior knowledge of the learners.

Secondly, accumulating evidence highlights the critical role of learners' prior knowledge for the cost-benefit ratio of collaborative learning (e.g., Janssen and Kirschner, 2020). This raises the question of how prior knowledge may influence the effects of constructive preparation activities on the exploitation of the potential collaboration benefits for in-depth knowledge acquisition. Research suggests that prior knowledge should facilitate the uptake and integration of co-learners' externalized knowledge and ideas encountered during collaboration. Yet, collaboration may become redundant when learners possess sufficient knowledge to deal with the learning requirements associated with the instructional material on their own (e.g., Nokes-Malach et al., 2012; Sears and Reagin, 2013; Retnowati et al., 2018; Zambrano et al., 2019). Hence, prior knowledge could be, on the one hand, too low to deal with the information processing demands and coordination costs associated with enacting interactive activities during collaboration. On the other hand, it could also be too high such that the learning requirements could be dealt with on one's own and, thus, making interactive activities unnecessary or their performance ineffective for learning (Nokes-Malach et al., 2012, 2015; Kirschner et al., 2018; cf. Janssen and Kirschner, 2020). This notion also receives indirect support from research on multimedia-learning, frequently evidencing the expertise reversal effect as a special case of the redundancy effect: if external information is presented that is already contained in a learner's long term memory, interferences may occur if ignoring the redundant information is difficult, thus inducing higher extraneous load (e.g., Sweller et al., 1998; Kalyuga et al., 2003; Janssen and Kirschner, 2020).

Consequently, whether the potential advantages or disadvantages of constructive preparation activities for interactive collaboration activities prevail may depend on prior knowledge (a-path-moderation, Figure 1B): constructive preparation activities may facilitate the subsequent execution of interactive activities (actor effect) for low prior knowledge learner's while being ineffective or even counterproductive for higher prior knowledge learners in this regard. Meanwhile, for the co-learner's constructive preparation activities to foster one's own interactive activities (partner effect), a certain amount of own prior knowledge may be necessary to deal with the associated costs. However, doing so may not pay off if one already possesses a relatively large body of prior knowledge. Such potential moderation effects through actor prior knowledge may also have consequences for the partner's interactive activities, for which the externalizations resulting from one's own interactive activities are an important input source (e.g., Chi, 2021).

Alternatively, or in addition, prior knowledge may also influence the effectiveness of interactive activities in view of deep comprehension outcomes (b-path-moderation, Figure 1B): previous work has argued that using the partner's externalizations to draw the inferences necessary to acquire a sound understanding of the instructional material (i.e., enacting interactive activities) may become less effective if prior knowledge is already sufficient to generate the inferences on one's own (e.g., Nokes-Malach et al., 2012; Deiglmayr and Schalk, 2015). In line with this, Mende et al. (2017) showed the positive effects of interactive activities on deep comprehension outcomes to diminish with increasing prior knowledge.

### 1.6 The present study

The overall purpose of the present study is to investigate whether performing constructive activities during individual preparation can help the individual learner to subsequently exploit the potential benefits of digital collaboration (i.e. CSCL) in terms of using the co-learner's externalized knowledge as resource for own inferencing processes (interactive activities) in the service of in-depth knowledge acquisition (deep comprehension outcomes). Thereby, our goals are two-fold:

The first goal consists in investigating how preparation tasks differently designed in terms of their generativity affect the execution of constructive preparation activities as a prerequisite for such beneficial collaboration processes and outcomes coming into effect. More specifically, we first aim to obtain a general picture of (a) which task and prior knowledge conditions are more or less beneficial for encouraging learners to execute constructive preparation activities, and (b) whether conditions that are more conducive in this regard also lead to more interactive collaboration activities and better deep comprehension outcomes. As a control, we also consider how preparation task generativity affects (a) the execution of constructive *collaboration* activities and (b) take not only the learners own but also the dyad partners' prior knowledge into account as potential moderator. Accordingly, our first two research questions (RQ) are as follows (see Figure 1A):

RQ 1a: What are the effects of preparation task generativity (i.e., low, moderate, high) on (a) the number of constructive preparation activities, (b) the number of constructive and interactive collaboration activities, and (c) deep comprehension in terms of transfer posttest achievement?

RQ 1b: Are these effects moderated by the actors and/or the partners' prior knowledge?

The second goal consists in investigating how and under what conditions whose constructive preparation activities influence the learner's personal exploitation of the potential collaboration benefits for in-depth knowledge acquisition. Accordingly, the main interest is in the potential indirect effects of the actors and the partner's constructive preparation activities on the *actor's* deep comprehension outcomes that are mediated via the *actor's* interactive collaboration activities and in whether such indirect effects may depend on the *actor's* prior knowledge. To obtain a comprehensive picture of the possible advantages and disadvantages of constructive preparation activities as well as the processes and conditions involved, but also for control purposes, we consider some more variables and effects. Specifically, we consider (a) not only the indirect but also the direct effects of constructive preparation activities explicitly and (b) the influences of the collaboration activities (mediators) on deep comprehension outcomes (b-paths) not only in terms of actor but also in terms of partner effects. Further, we (c) control for constructive activities carried out during collaboration as potential alternative mediator, and (d) include not only the actor's but also the partner's prior knowledge as potential moderator to more exhaustively capture the conditions that may play a role in the reciprocal influence processes between the learners. Thus, our second two RQ's are as follows (see Figure 1B):

RQ 2a: Considering constructive and interactive collaboration activities as potential mediators, what are the direct and indirect actor and/or partner effects of constructive preparation activities on deep comprehension outcomes?

RQ 2b: Are these effects moderated by the actor's and/or the partner's prior knowledge?

Given the resulting moderated mediation model, four kinds of (moderated) indirect effects might occur per mediator when considering both the a-paths and the b-paths in terms of actor and partner effects (e.g., Sadler et al., 2011): actor-actor-effects, actor-partner-effects, partner-actor-effects, and partner-partner effects.

## 2 Materials and methods

### 2.1 Participants and setting

Due to the complexity associated with power calculations for CSCL experiments, there are no established guidelines to date (Janssen and Kollar, 2021). Therefore, we based our sample size on previous, comparable studies (e.g., Deiglmayr and Schalk, 2015; Jurkowski and Hänze, 2015; Vogel et al., 2016; Lam and Muldner, 2017; Tsovaltzi et al., 2017). Consequently, we conducted an experiment in which a total of 138 students (69 dyads) from a German university went through a CSCL scenario on the human circulatory system. Excluded from participation were students of medicine, biology, or similar fields, as well as non-native speakers. Some students were dyad-wise excluded *post hoc* because they did not follow the instructions in the learning phase (5 dyads), the data were incomplete (1 dyad), a dyad member turned out to be a non-native speaker (1 dyad) or due to technical problems (1 dyad). The final sample contained 61 dyads with a total of 122 undergraduate students (72.9 % female, mean age: 22.81 years, *SD* = 3.95) of psychology (49.2%) and educational sciences (50.8%).

### 2.2 Learning material

As learning material, we used an expository text on the human circulatory system translated and adapted from Chi et al. (2001). The text consisted of 1,090 words, approximately evenly distributed over 3 sections entitled as “The heart,” “The vessels,” and “The subsystems of the circulatory system.” The text was presented on the monitor throughout the learning phase within the CSCL environment. A sound comprehension of the circulatory system requires not only knowledge of its single components and their properties, but also an understanding of the coordinated interaction between these components at different hierarchy levels and how these interactions provide the vital functions of the system as a whole. An expository text—such as the one used in this study—typically leaves out many of these features, relationships and interactions and, thus, leaves a lot of room for interpretation on the part of the learners. In other words, inferences are necessary to fill in these gaps and to build a proper mental model of the system that enables the flexible application of what has been studied (Chi et al., 1994).

### 2.3 Design and procedure

Participants arrived at the lab, were greeted and assigned to their computer desks. After an introduction to the CSCL environment, participants' demographic data and prior knowledge were obtained through an electronic pretest. All participants were informed that they would be learning about the human circulatory system with a text. They were instructed to develop an understanding of the circulatory system in terms of how it is composed, how it functions, and what its general purpose is (see Jeong and Chi, 2007).

**Figure 2:**
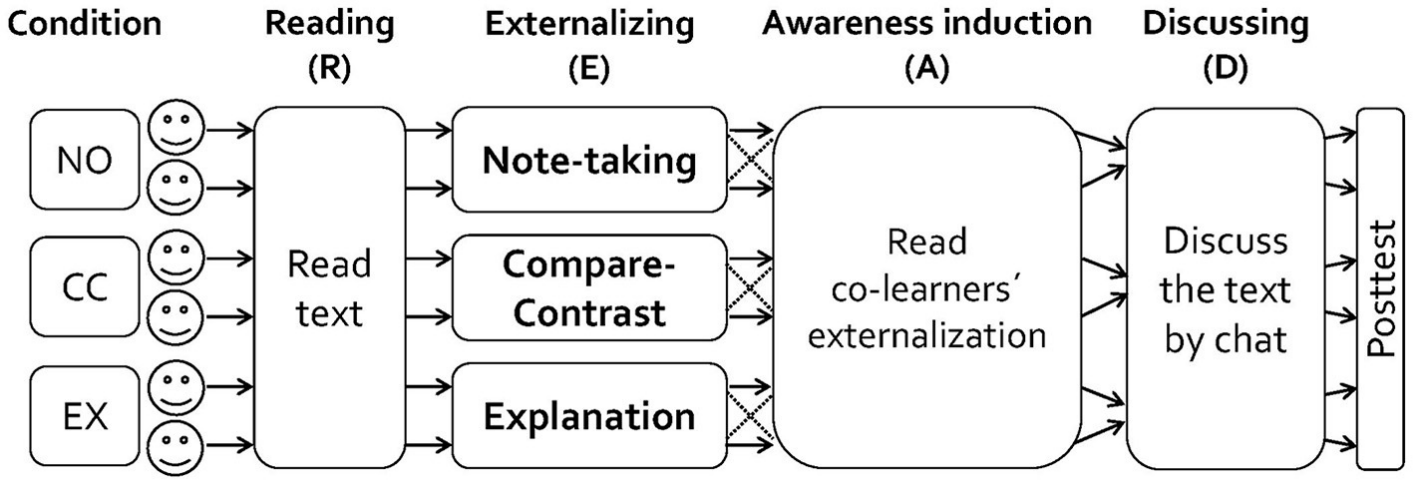
Experimental design and procedure. NO, CC, and EX refer to the note-taking, compare-contrast, and explanation task conditions, respectively.

Subsequently, the students were randomly grouped into stable dyads automatically by the CSCL system. All dyads followed a CSCL-script developed by the authors (READ-script; Mende et al., 2017) which prescribed the following learning phases (see Figure 2).

After reading the whole text (reading phase), each learner worked individually on a task and wrote their answer in a text box (externalization-phase). These task responses were subsequently delivered to the co-learners (i.e., dyad partners) by the CSCL system and both learners were explicitly requested to inspect each other's task responses (awareness induction phase). Finally, learners were asked to collaboratively discuss the text using the chat function that was now available (discussion phase). Here, they received the instruction to collaborate in order to help each other in improving understanding. The previously produced individual externalizations of both learners were still available to everyone during this phase.

The externalization, awareness induction, and discussion phases were repeated for each of the three text sections. In each of the externalization phases, participants were given a section-specific task and the text was automatically scrolled to the beginning of the relevant section. Depending on experimental condition, the dyads were randomly assigned to either one of three versions of the described script which differed only with respect to the type of preparation task applied in the individual externalization phases (see Appendix A in the Supplementary material).

Participants in the note-taking task condition were not specifically requested to perform constructive activities. Subjects in the compare-contrast task condition were required to compare-and-contrast central concepts addressed by the text. For example, subjects were asked to compare the different kinds of blood vessels of the circulatory system regarding their components and the processes they are involved in. Since many of these similarity and difference relations were not explicitly stated in the text, learners had to infer them, typically by connecting different information that are explicitly given in the text. In other words, learners had to perform constructive activities to complete this type of task. Participants in the explanation task condition were required to provide explanations related to the same central text concepts as in the compare-contrast task condition. For example, the learners were asked to find reasons why our circulatory system entails different types of blood vessels instead of only one type. To this end, the learners had not only to infer comparative relations, but also to connect these relations with each other in order to formulate explanations for the existence of the components addressed in the respective task. Besides of connecting different text information, this required to insert general or domain specific prior knowledge. In other words, compared to the compare-contrast tasks, learners had to perform even more constructive activities in order to complete the explanation tasks. Taken together, the extent and explicitness to which the described tasks ask for the execution of constructive activities (i.e., the task generativity) increases from note-taking (low) to compare-contrast (moderate) to explanation (high).

To keep learning time constant between experimental conditions, subjects were given a target time of 10 min each for the externalization and the discussion phases, being allowed to proceed to the next phase after 8 min at the earliest, and automatically forwarded after 12 min at the latest. One week after the treatment participants reentered the lab to answer a posttest capturing their text comprehension.

### 2.4 Measures

#### 2.4.1 Pretest

We assessed the participants' prior knowledge with a test adapted from Jeong and Chi (2007). Students were requested to describe the blood path of the circulatory system in a textbox. They were asked to do this in as much detail as they could, while also including components and processes that play a role in the human circulatory system.

To code participants' prior knowledge, a predefined template was used that included topic-relevant idea units in terms of knowledge pieces about the circulatory system, for instance, “blood moves from the heart to the body” or “the heart is a pump.” Participants received one point for each piece of knowledge expressed (Jeong and Chi, 2007). A second rater coded 17% of the data for inter-rater reliability (α_Krippendorf_ = 0.90). The resulting prior knowledge score represents the sum of knowledge pieces contained in a participant's written response to the pretest. Please note that this score does not include information on the relationships among the idea units or learners' mental model about the circulatory system.

#### 2.4.2 Coding of learners' individual externalizations

In order to assess the extent to which the learners enacted constructive preparation activities, the individual externalizations were subjected to a coding procedure. More concretely, the quality of participants' responses to the preparation tasks were coded using a scheme developed by the authors (Mende et al., 2017) based on previously published operationalizations of constructive activities (e.g., Chi and Wylie, 2014; De Backer et al., 2014; Roscoe, 2014). To this end, we assessed the occurrence of constructive activity indicators at the protocol level in terms of the number of sentences containing inferences, that is, topic-relevant information not already given in the learning text. This can have the form of, for instance, comparing the thickness of arteries and capillaries or generating a causal explanation such as “due to their thick walls, diffusion is not possible in the arteries” since these comparisons and explanations were not explicitly presented in the text. By contrast, mere repetitions of text information were not considered constructive activity.

By means of the described procedure, each of the three externalizations per participant were evaluated with respect to the number of constructive activities. A second rater coded 25% of the individual externalization protocols (α_Krippendorf_ = 0.91). The resulting score represents the sum of constructive preparation activities a learner has performed during the individual externalization phases.

#### 2.4.3 Coding of the collaborative discussion activities

In order to assess the extent to which the learners performed constructive and interactive activities during the collaborative discussion phases, the quality of the chat dialogues was subjected to a coding procedure. To this end, we applied a previously developed coding scheme (Mende et al., 2017) that has been adapted from previous work (e.g., Jeong and Chi, 2007; Berkowitz et al., 2008; Noroozi et al., 2013; Chi and Wylie, 2014; De Backer et al., 2014; Roscoe, 2014).

Participants' chat messages were first segmented according to punctuation and “connectives” (Strijbos and Stahl, 2007; Erkens and Janssen, 2008). In a second step, each segment was assessed for whether it contains topic-relevant information (i.e., information about the circulatory system; α_Krippendorf_ = 0.97). This was done because computer-based learning dialogues typically comprise not only utterances directly related to processing the learning content but also utterances related to purely metacognitive, technical, coordinative or social concerns (e.g., Paulus, 2009; De Backer et al., 2014). Only segments containing topic-relevant information (e.g., “The heart is divided into four chambers, right?”; “I think that blood is oxygenated in the lungs”) were considered for further coding. The remaining segments (e.g., “I understood the text passage well,” “What should we talk about next?,” “Which button do I need to press to continue?,” and “What will we have for lunch?”) were excluded from further analyses.

In a third step, two independent decisions were made for each topic-related segment: (a) does the segment contain an inference (see above)? (b) does the segment contain indications of referencing to a prior contribution of the co-learner in terms of taking up or incorporating information expressed in the dyad partner's individual externalization or previous chat messages? A second rater coded 25% of the discussion protocols (α_Krippendorf_ = 0.82–0.88). Segments containing an inference without indications of referencing were counted as constructive activity and segments containing an inference with indications of referencing were counted as interactive activity. Segments containing no inference were not considered for further analyses. The resulting scores represent the sum of constructive or interactive activities a learner has performed during the collaborative discussion phases.

#### 2.4.4 Posttest

One week after the treatment participants were administered with a computer-based posttest adapted from Chi et al. (2001) that assessed their knowledge about different aspects of the human circulatory system comprising the components, functioning and purposes of the heart, the vessels, and the different sub-circuits. The test consisted of 30 multiple choice questions covering shallow and deep text comprehension. Each question consisted of four answer options, with only one option being correct. Since retest effects can arise in pre-post-test designs, the multiple-choice format was only used in the posttest while the open response task format was used in the pretest.

The shallow comprehension subtest included twenty questions that could be answered by either restating an information explicitly provided in the learning material or by combining information which were explicitly given across several sentences of the learning material. The average item difficulty was 0.59 (*SD* = 0.14) and ranged from 0.40 to 0.90.

To correctly answer the 10 questions forming the deep comprehension subtest, learners had to transfer the text information to issues not directly addressed within the sentences contained in the learning material. That is, answering this kind of questions required that the learners had integrated their prior knowledge with the text information and formed a proper mental model of the circulatory system (Chi et al., 2001). The average item difficulty was 0.42 (*SD* = 0.21) and ranged from 0.16 to 0.75.

Examples of the items and answer options are provided in Appendix B in the Supplementary material. For each participant we computed percentages of correctly answered questions per subtest. Please note that the focus of this work is on learners' deep comprehension. Therefore, the results of the shallow comprehension test are only included in the descriptive statistics for overview purposes.

### 2.5 Data analysis

Since subjects were nested in dyads, we conducted linear mixed regression analyses for dyadic data (Kenny et al., 2006), using the restricted maximum likelihood method (REML) for effect estimations and the maximum likelihood method (ML) for assessment of model fit changes in terms of likelihood ratio tests (e.g., Campbell and Kashy, 2002).

As some of our research variables revealed deviations from a normal distribution, we performed bias-corrected and accelerated bootstrap analyses with 5,000 resamples to estimate the standard errors and confidence intervals for all regression coefficients (e.g., Puth et al., 2015; Scharkow, 2017). To be considered significant at the 5% significance level, an effect must not include zero in the 95% bootstrap interval. We centered all continuous predictors before analyses. Unstandardized regression coefficients are reported.

#### 2.5.1 Research question 1

RQ1 investigated the effects of three individual preparation tasks differing in their generativity, as well as the moderating role of actor's and partner's prior knowledge in view of the number of constructive preparation activities, constructive and interactive collaboration activities as well as deep comprehension posttest achievement. Moderated mixed regressions were performed for each dependent variable applying a two-step approach: In a first step, experimental condition as well as the actor's and partner's prior knowledge were entered into the regression. Experimental condition was dummy-coded so that the compare-contrast task condition and the explanation task condition were each compared with the note-taking task condition as the reference group. In a second step, we entered the two-way interaction terms between condition and prior knowledge variables.

In order to also capture the effect of the explanation task condition in relation to the compare-contrast task condition, this two-step procedure was repeated using sequential coding. That is, this time the explanation task condition was compared with the compare-contrast task condition and the latter again with the note-taking task condition.

A moderator effect was assumed if the addition of the interaction terms in step 2 resulted in a significant improvement in model fit and the corresponding interaction term had a significant regression weight. In such a case, the simple slopes for the effects of task type were calculated at different values of prior knowledge as a follow-up analysis: at the 10^th^, 25^th^, 50^th^, 75^th^, and the 90^th^ percentile of the sample distribution (e.g., Hayes, 2013), which correspond to prior knowledge scores of 4.00, 7.00, 11.50, 17.00, and 22.00.

#### 2.5.2 Research question 2

Research question 2 was concerned with potential indirect actor and partner effects of constructive preparation activities (independent variables) on deep comprehension outcomes (dependent variable) through constructive and interactive collaboration activities (mediators) as well as the moderating role of prior knowledge for such indirect effects. In addition, we considered the direct actor and partner effects of constructive preparation activities on deep comprehension outcomes that were explicitly not transmitted through the mentioned mediators.

To investigate research question 2, moderated mediation analyses were conducted (e.g., Hayes, 2013; Song, 2018). Thereby we applied the procedure for estimating an Actor-Partner-Interdependence model for indistinguishable dyads (Kenny et al., 2006).

To assess the occurrence of indirect effects we followed the approach of Yzerbyt et al. (2018). The authors recommend testing three effects in sequence, all of which must be statistically significant to conclude that there is an indirect effect. These include a-path analysis, b-path analysis, and a^*^b-path analysis, with the latter being used to estimate the actual indirect effect (e.g., Hayes, 2013):

First, in the course of the a-path analyses we examined the actor and partner effects of constructive preparation activities (independent variables) on constructive and interactive collaboration activities (mediators) and whether these effects are moderated by the actor's and/or the partner's prior knowledge. Within the a-path analyses, moderation was assessed following the two-step procedure already described regarding the analyses for research question 1. If neither a significant (moderated) actor nor a partner effect was found in view of a mediator, the latter was not further subjected to the following b-path analyses.

Second, in the course of the b-path analysis, while controlling for actor and partner effects of constructive preparation activities (independent variables), we examined the actor and partner effects of the mediators not excluded during a-path analyses on deep comprehension posttest achievement (dependent variable) and whether these effects are moderated by the actor's and/or the partner's prior knowledge. If neither a significant (moderated) actor nor a partner effect of a mediator on a dependent variable was found, the mediator was not further subjected to the following a^*^b-path analyses. Within the b-path analysis model, also the direct actor and partner effects (c') can be obtained in terms of the effects of the constructive preparation activities controlled for the effects of the potential mediators.

Third, potential mediators not excluded during the previous steps were subjected to the a^*^b-path analyses. To this end, the respective a-path and the b-path coefficients as well as their bootstrapped standard errors were used to calculate the indirect effects (a^*^b-paths) along with 95% Monte Carlo confidence intervals based on 100,000 replications, using the SPSS macro MCMED (Hayes, 2013). If an indirect effect included an a-path and/or b-path coefficient for which the previous analyses indicated significant moderation, the respective a-path and/or b-path coefficients at different moderator values (10^th^, 25^th^, 50^th^, 75^th^, and 90^th^ percentile) were used to calculate the indirect effect, resulting in a total of five estimates of the respective indirect effect (e.g., Hayes, 2013).

## 3 Results

### 3.1 Descriptive and preliminary analyses

Table 1 summarizes descriptive statistics for pretest variables. No significant pre-group differences regarding sex [$X_{\left(2,\text{N}=122\right)}^{2}$ = 1.33, *n.s*.] subject of study [$X_{\left(2,\text{N}=122\right)}^{2}$ = 2.42, *n.s*], age [*F*_(2, 121)_ = 1.42, *n.s*.], or prior knowledge [*F*_(2, 121)_ = 0.65, *n.s*.] occurred.

**Table 1:** Descriptives (*N* = 122).

**Experimental condition (task generativity)**	**Low: note-taking (*n* = 36)**	**Moderate: compare-contrast (*n* = 42)**	**High: explanation (*n* = 44)**
Age: *M* (*SD*)	22.19 (3.11)	22.52 (3.47)	23.59 (4.85)
Prior knowledge: *M* (*SD*)	13.08 (6.69)	11.50 (6.18)	13.05 (8.39)
Shallow comprehension	61.53 (16.60)	60.71 (18.10)	54.77 (13.47)
post-test^a^: *M* (*SD*)			
**Sex: percentages**			
Female	75%	66.7%	77.3%
Male	25%	33.3%	22.7%
**Subject of study: percentages**			
Psychology	58.3%	50.0%	40.9%
Educational Sciences	41.7%	50.0%	59.1%

^a^ Percent of MC items answered correctly.

### 3.2 RQ1: effects of preparation task generativity and the potentially moderating role of prior knowledge

Statistical values are not presented in the text for better readability. The results of the analyses are provided in detail in Appendix C in the Supplementary material. An overview is given in Table 2. In the following, results are addressed separately for the different dependent variables.

**Table 2:** Descriptives of dependent variables and overview of the results concerning RQ1 (*N* = 122).

**Experimental condition (task generativity)**	**Low: note-taking (*****n*** = **36)**		**Moderate: compare-contrast (*****n*** = **42)**		**High: explanation (*****n*** = **44)**		**Effect: experimental comparison^b^**
**Dependent variable**	***M***	***SD***	***M***	***SD***	***M***	***SD***	
Constructive preparation activities	1.83	1.81	7.88	4.82	13.00	5.33	Low vs. moderate: see Figure 3^c^ Low vs. high: see Figure 3^c^ Moderate vs. high: 4.59^*^
Constructive collaboration activities	2.11	1.94	1.74	1.96	1.34	1.38	Low vs. moderate: −0.38 Low vs. high: −0.77^*^ Moderate vs. high: −0.39
Interactive collaboration activities	4.75	3.75	4.45	3.88	5.55	4.74	Low vs. moderate: −0.05 Low vs. high: 0.80 Moderate vs. high: 0.85
Deep comprehension posttest^a^	45.56	16.29	36.19	17.52	44.31	18.85	Low vs. moderate: −7.47^*^ Low vs. high: −1.19 Moderate vs. high: 6.28^*^

^a^Percent of MC items answered correctly. ^b^Unstandardized regression coefficients are reported as obtained from the results of the mixed moderated regression analyses; see Appendix C in the Supplementary material for details. ^c^ The effect is moderated by the actor's prior knowledge and therefore detailed in Figure 3. ^*^p < 0.05 as determined by the 95% bias corrected and accelerated bootstrap confidence intervals.

#### 3.2.1 Constructive preparation activities

**Figure 3:**
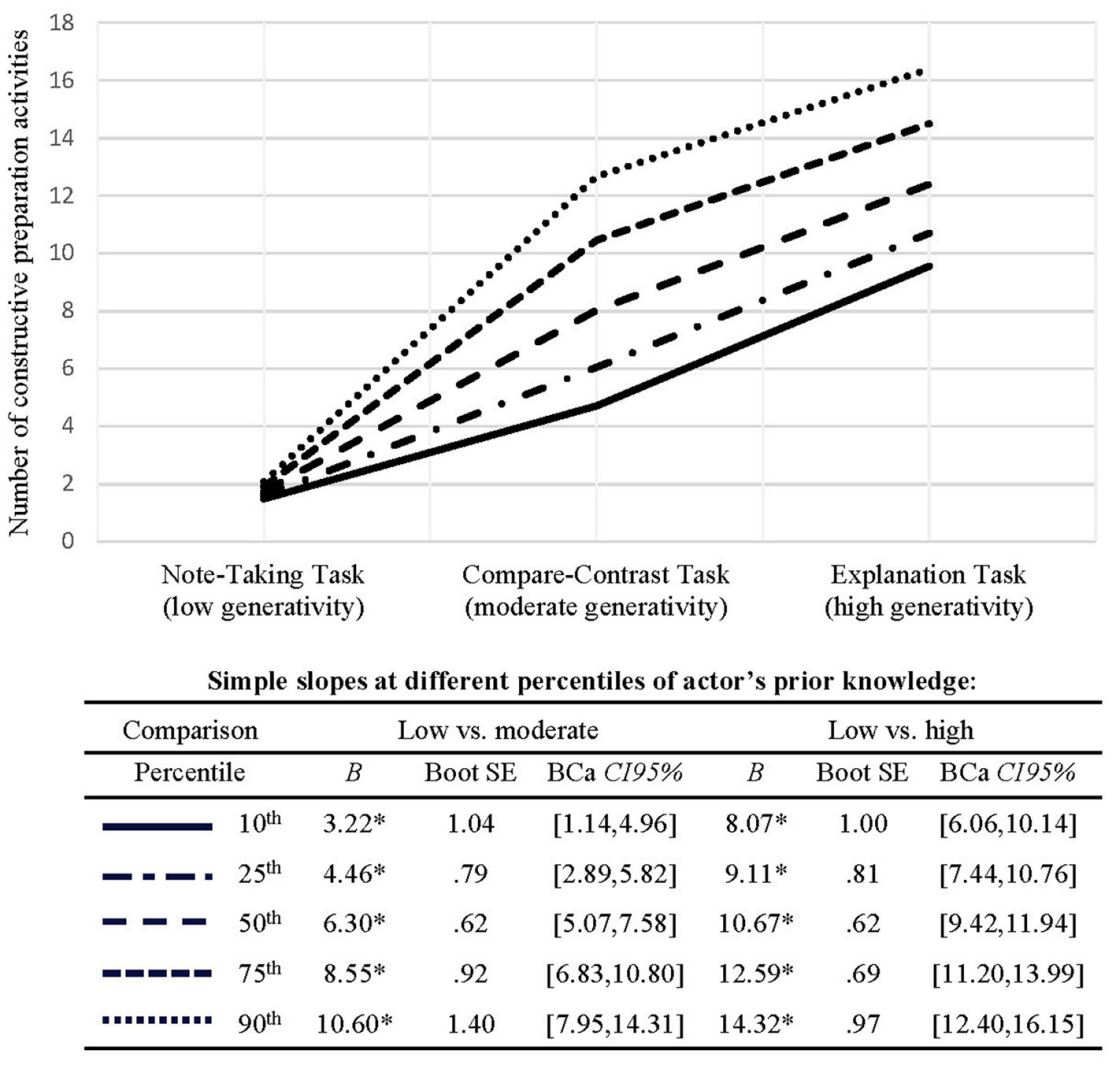
Follow-up-analyses of the significant interaction effects between task type and actor's prior knowledge on self-performed constructive preparation activities. The respective effects are visualized and reported in terms of simple slopes, consonant with the actor's prior knowledge at the 10^th^, 25^th^, 50^th^, 75^th^, and 90^th^ percentile of the distribution. Unstandardized regression weights are reported. All continuous predictors were centered prior to the analyses. Asterisks indicate significance at the 5% level as determined by the 95% bias corrected and accelerated bootstrap confidence intervals.

Both, the compare-contrast and the explanation tasks led the learners to perform more constructive preparation activities than the note-taking tasks. The actor's prior knowledge moderated these positive main effects, though without qualifying them (see Table 2, first row & Figure 3). That is, the compare-contrast and the explanation tasks each had a significant positive effect on the constructive preparation activities of all learners, but these effects were stronger for learners with higher prior knowledge in both cases as indicated by the simple slope tests (see Figure 3).

In addition, findings revealed the explanation tasks to be superior to the compare-contrast tasks in terms of inducing constructive preparation activities. For this effect, the results showed no indications of a moderating role of the actor's or the partner's prior knowledge.

#### 3.2.2 Constructive collaboration activities

The explanation task led learners to perform less constructive collaboration activities than the note-taking task (see Table 2, second row). No other main effects or any moderation effects were found.

#### 3.2.3 Interactive collaboration activities

No main or moderated effects of task generativity were found in view of interactive collaboration activities (see Table 2, third row).

#### 3.2.4 Deep comprehension achievement

In terms of deep comprehension posttest achievement, the compare-contrast task condition participants performed significantly worse than the subjects in the note-taking and explanation task conditions (see Table 2, fourth row). This effect was neither moderated by the actor's nor partner's prior knowledge. No further main or any moderation effects were found.

### 3.3 RQ2: indirect and direct actor and partner effects of constructive preparation activities and the potentially moderating role of prior knowledge

**Figure 4:**
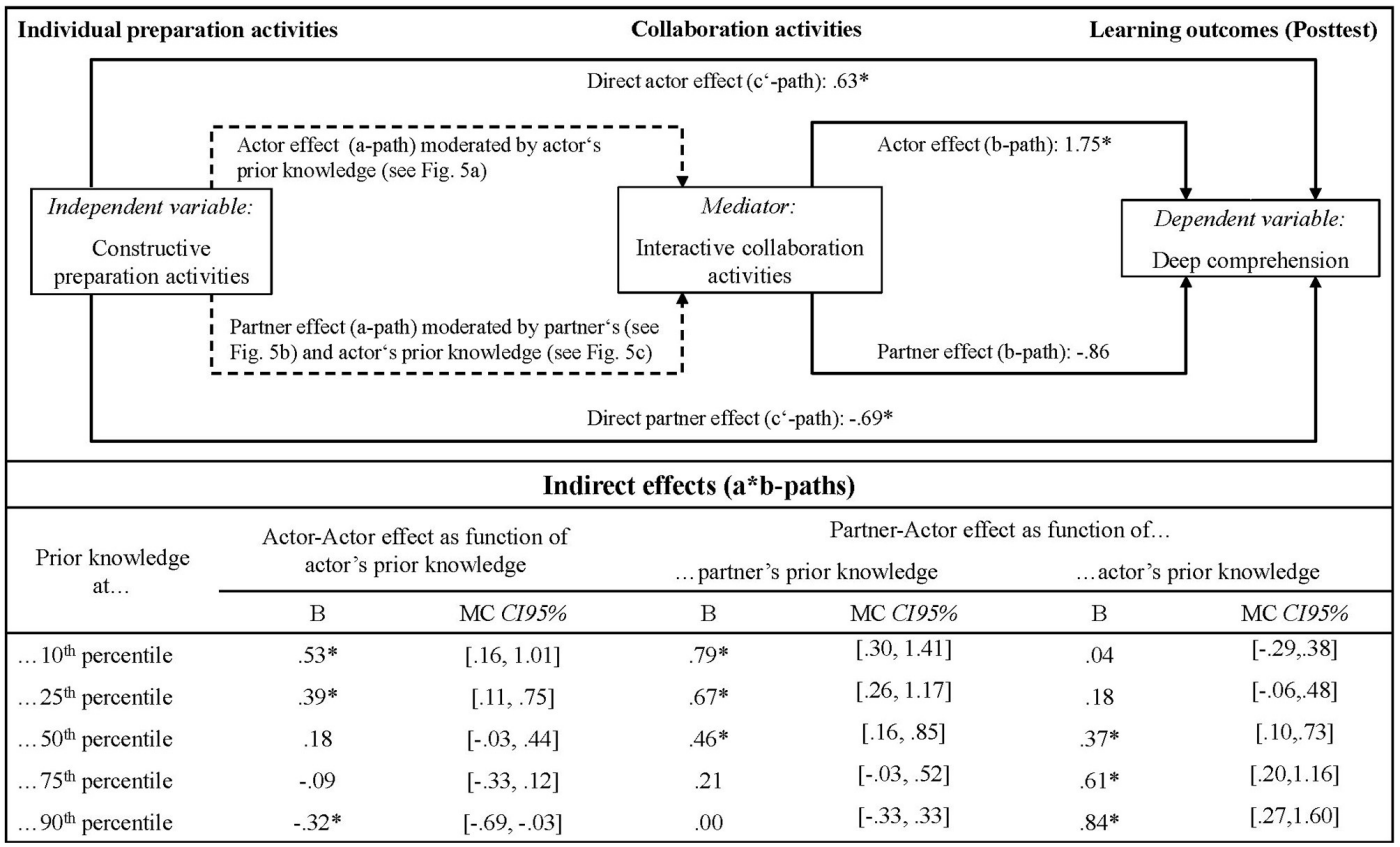
Summary of the moderated mediation analyses (RQ 2). Only interactive collaboration activities are presented as mediator and included in tests for indirect effects, as constructive collaboration activities have been excluded as potential mediator during the preceding a-path and b-path analyses; see Appendix D in the Supplementary material for details. Unmoderated paths are indicated by solid lines and labeled with the according main effect. Moderated paths are indicated by dotted lines with the respective effects being detailed in Figure 5. Since all to be estimated a*b-paths include an a-path coefficient which has been indicated to be moderated by the actor's and/or the partner's prior knowledge, each indirect effect is reported in terms of simple slopes consonant with prior knowledge at the 10^th^, 25^th^, 50^th^, 75^th^, and 90^th^ percentile of the distribution. Unstandardized regression weights are reported. All continuous predictors were centered prior to the analyses. Asterisks indicate significance at the 5% level as determined by the 95% bias corrected and accelerated bootstrap confidence intervals or, in case of indirect effects, the 95% Monte Carlo confidence intervals.

The results of the a-path and b-path analyses are provided in detail in Appendix D in the Supplementary material. An overview is given in Figure 4 together with the results of the final a^*^b-path tests for (moderated) indirect effects. In the following, results are addressed separately for the different mediators and the direct effects.

#### 3.3.1 Constructive collaboration activities as potential mediator

Constructive collaboration activities were excluded as potential mediator already during a-path analyses since neither actor nor partner effects, whether unmoderated or moderated by the actor's or the partner's prior knowledge, were observed (see Appendix D in the Supplementary material).

#### 3.3.2 Interactive collaboration activities as potential mediator

**Figure 5:**
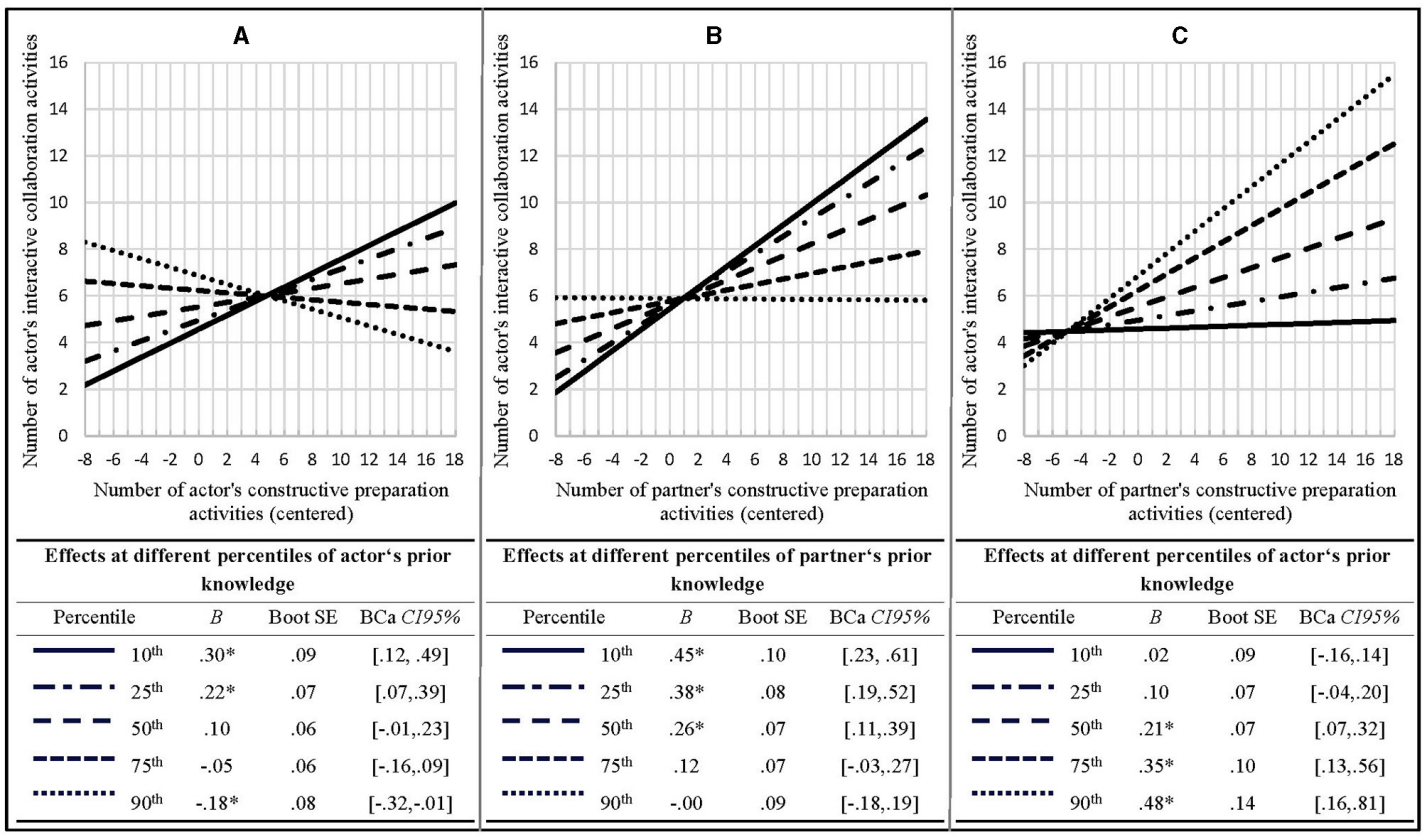
Follow-up-analyses of the significant interaction effects between constructive preparation activities and prior knowledge on self-performed interactive collaboration activities. **(A)** Actor effect of constructive preparation activities on interactive collaboration activities as function of actor's prior knowledge. **(B)** Partner effect of constructive preparation activities on interactive collaboration activities as function of partner's prior knowledge. **(C)** Partner effect of constructive preparation activities on interactive collaboration activities as function of actor's prior knowledge. The respective effects are visualized and reported in terms of simple slopes, consonant with prior knowledge at the 10^th^, 25^th^, 50^th^, 75^th^, and 90^th^ percentile of the distribution. Unstandardized regression weights are reported. All continuous predictors were centered prior to the analyses. Asterisks indicate significance at the 5% level as determined by the 95% bias corrected and accelerated bootstrap confidence intervals.

First, an indirect actor-actor effect moderated by the actor's prior knowledge was found: mediated via self-performed interactive collaboration activities, learners with relatively low prior knowledge (10th and 25th percentile) benefitted from performing constructive preparation activities in terms of deep comprehension outcomes while learners with relatively high prior knowledge (90th percentile) suffered losses from performing constructive preparation activities (see Figure 4 lower part). Consulting the results of the a-path and b-path analyses (Figure 4 upper part) helps interpreting this effect: self-performed constructive preparation activities fostered the learner's execution of interactive collaboration activities when their own prior knowledge was relatively low (10^th^ and 25^th^ percentile) but reduced the execution of interactive activities when prior knowledge was relatively high (90th percentile; see Figure 5A). The self-performed interactive activities in turn promoted own deep comprehension outcomes for all learners irrespective of prior knowledge (Figure 4 upper part). Hence, for learners with relatively high prior knowledge the execution of constructive preparation activities was detrimental to their deep comprehension outcomes as far as these activities prevented them from enacting interactive collaboration activities.

Second, we obtained an indirect partner-actor effect which was moderated by the partner's prior knowledge. Mediated via self-performed interactive collaboration activities, learners benefitted in terms of deep comprehension from the constructive preparation activities of their co-learners, but only if the latter's prior knowledge was relatively low to moderate (10^th^, 25^th^, and 50^th^ percentile; Figure 4 lower part). Consulting the a-path and b-path analyses results shows relatively low to moderate (10^th^, 25^th^, and 50^th^ percentile) but not higher prior knowledge co-learner's constructive preparation activities fostered one's own interactive collaboration activities (Figure 5B). The latter in turn promoted one's own deep comprehension outcomes regardless of prior knowledge (Figure 4 upper part). Put another way, the self-performed constructive preparation activities of learners with relatively low to moderate prior knowledge had a positive indirect effect on their co-learner's deep comprehension outcomes mediated by their co-learner's interactive activities.

Third, we found the indirect partner-actor-effect just described to be also moderated by the actor's prior knowledge. Mediated via their own interactive collaboration activities, learners with moderate to relatively high prior knowledge (50^th^, 75^th^, and 90^th^ percentile) benefitted from their co-learners enacted constructive preparation activities in terms of deep comprehension (Figure 4 lower part). Consulting a-path and b-path analyses reveals moderate to relatively high (50^th^, 75^th^, and 90^th^ percentile) but not relatively low prior knowledge learners' performance of interactive activities was positively affected by their co-learners previously executed constructive preparation activities (Figure 5C). Executing interactive activities in turn promoted own deep comprehension outcomes irrespective of prior knowledge (Figure 4 upper part).

#### 3.3.3 Direct effect of constructive preparation activities

Constructive preparation activities were also found to have direct actor and partner effects on deep comprehension that are not mediated by interactive collaboration activities (Figure 4 upper part): The performance of constructive preparation activities had a positive direct influence on the learner's own deep comprehension in the sense of an actor effect. In contrast, co-learners' constructive preparation activities directly impaired learner's deep comprehension in terms of a negative partner effect. No indications of moderation through prior knowledge were obtained in either case.

## 4 Discussion

The aim of this study was to investigate whether performing constructive activities during individual preparation can help learners to subsequently exploit the potential benefits of digital collaboration in terms of using co-learners externalized knowledge as resource for own inferencing processes (interactive activities) in the service of in-depth knowledge acquisition (deep comprehension outcomes). To address this aim, we firstly investigated on a more general level whether more rather than less generative tasks intended to induce constructive preparation activities are suited to increase the execution of interactive activities and deep comprehension achievement (RQ1). We secondly examined on a more detailed level the direct and indirect (via interactive activities) effects of the actor's and partner's constructive preparation activities on deep comprehension outcomes (RQ2). Prior knowledge was considered as potential moderator in both cases.

Overall, the results in response to RQ1 suggest increasing the preparation task generativity to be an effective way to raise the number of constructive preparation activities executed by the individual learning-dyad members prior to collaboration. However, the results further indicate that this *per se* is not sufficient to lead learners to better utilize subsequent collaboration in terms of in-depth knowledge acquisition. The analyses conducted in the course of addressing RQ2 provided some insights into the possible reasons for this pattern of results. These findings suggest that the execution of constructive activities during an individual preparation yields not only advantages but also disadvantages in view of subsequent collaborative learning: though self-performed constructive preparation activities had direct benefits for own deep comprehension outcomes, they promoted deep learning *from* subsequent collaboration only for learners with relatively low prior knowledge while they were ineffective or even detrimental in this regard for learners with relatively high prior knowledge. Co-learners' constructive preparation activities fostered one's own deep learning from collaboration under specific conditions of own and partner's prior knowledge, but negatively affected one's deep comprehension outcomes on the direct path. In other words, given the present findings, the answer to the question of whether constructive preparation activities can promote deep learning from subsequent collaboration seems to be an “it depends.” In the following we discuss the results related to RQ1 and RQ2 in more detail.

### 4.1 The antecedents and consequences of constructive preparation activities

Concerning RQ1, our results revealed that more generative tasks led the learners to execute more constructive preparation activities during individual preparation: the number of constructive preparation activities significantly increased from the note-taking task (low generativity) over the compare-contrast task (moderate generativity) to the explanation task (high generativity). This is in line with previous generative learning research on the effectiveness of these different tasks in terms of inducing deep learning processes (e.g., Grabowski, 2004; Sidney et al., 2015; Chin et al., 2016; Ponce et al., 2020). Furthermore, in reference to the low generative task, the positive effects of the more generative tasks (i.e., compare-contrast and explanation) were the more pronounced, the higher the learners' prior knowledge which has been also expected in the face of previous research on text comprehension and cognitive load (e.g., Kintsch, 1998; Best et al., 2005; Chen et al., 2017). Taken together, a higher preparation task generativity consistently led all learners to enact more constructive preparation activities, although the effects were stronger for more knowledgeable learners.

This positive task generativity effects did, however, not transfer to the number of interactive (or constructive) collaboration activities and deep comprehension outcomes. We even observed participants in the compare-contrast task condition to perform significantly worse than the subjects in the note-taking and the explanation task conditions in terms of deep comprehension outcomes as indicated by the transfer posttest. As a possible explanation, the compare-contrast task may have focused the learners too much on single comparisons between the circulatory system components, thus preventing them from developing a more comprehensive understanding of how the system works as a whole, resulting in poorer transfer achievement (e.g., Chin et al., 2016). As a related explanation, the deep comprehension posttest primarily captured learners understanding of the functioning of the circulatory system in terms of cause-effect-relations. Thus, the fit between the preparatory compare-contrast task and the posttest was relatively low compared to the other conditions.

To summarize, in the present study, a higher preparation task generativity led learners to execute a greater number of constructive preparation activities. However, consistent with previous studies (e.g., Lam and Kapur, 2017; Lam and Muldner, 2017; Lam, 2019), we found no evidence that tasks of higher generativity are better suited than tasks of lower generativity to help learners take advantage of the potential benefits of subsequent collaboration for in-depth knowledge acquisition.

This invites a closer look into the mechanisms involved and the conditions relevant for the effects of constructive preparation activities enacted by oneself and one's co-learner (RQ2). To first give a general overview: both, one's own and one's partner's constructive preparation activities were found to have significant direct as well as moderated indirect effects on one's own deep comprehension outcomes. These indirect effects all included self-performed interactive collaboration activities as mediator. Specifically, learners own interactive activities positively affected their own deep comprehension outcomes irrespective of prior knowledge. *Moderated* indirect effects of one's own as well as the partner's constructive preparation activities occurred because each affected the execution of interactive activities differently in dependence of own and/or the partner's prior knowledge. In the following we discuss these indirect effects along with the direct effects.

To begin with, self-performed constructive preparation activities in themselves already fostered own deep comprehension outcomes directly, that is, not mediated by interactive (or constructive) collaboration activities. This could be expected in light of previous findings on the positive effects of constructive activities on deep learning (e.g., Chi and Wylie, 2014). Extending previous research, our results also show that executing constructive activities during an individual preparation can, in the sense of an indirect effect, promote but also hinder one's execution of interactive activities and, in turn, deep comprehension outcomes depending on own prior knowledge (actor-actor effect moderated by actor's prior knowledge). More specifically, our findings suggest that self-performed constructive preparation activities promote one's own subsequent execution of interactive collaboration activities at relatively low prior knowledge levels, have no effect at higher levels, and even lead to less interactive activities at the highest level considered (i.e., 90^th^ percentile). This is in line with the assumptions of benefit-cost approaches on the role of prior knowledge on collaborative learning (e.g., Nokes-Malach et al., 2012, 2015; Janssen and Kirschner, 2020): building relevant knowledge structures first through constructive preparation activities seems to have helped learners with little prior knowledge to deal with the costs associated with taking up and integrating externalized knowledge from co-learners (i.e., performing interactive activities) during collaboration while still leaving enough room to experience benefits from doing so, for instance, in terms of further developing or differentiating own initial conclusions and ideas together with the co-learner during discussion. Learners who already possessed a larger body of relevant prior knowledge were possibly more capable to deal with the costs of interactive activities from the outset, so that the execution of constructive preparation activities had no added value for them in this regard. Moreover, the higher the prior knowledge, the more likely the execution of constructive activities might have led learners to come to a comprehensive understanding of the instructional material already at the end of the preparation. This might have reduced the expected potential benefits of interactive activities and, thus, their execution. The results indicate, however, that self-performed interactive activities were conducive to deep comprehension outcomes irrespective of prior knowledge. That is, also high prior knowledge learners benefitted from enacting interactive activities in the present study.

The described prior knowledge dependency of the effects of one's own constructive preparation activities on one's own interactive activities seems to have consequences for the co-learner with respect to his or her usage of the potential collaboration benefits in terms of in-depth knowledge acquisition as well. More specifically, constructive preparation activities executed by learners at relatively lower levels of prior knowledge did not only had a positive indirect effect on their own deep comprehension outcomes via their own interactive activities (actor-actor effect moderated by the actor's prior knowledge) but also a positive indirect effect on their partner's deep comprehension via their partner's interactive activities (partner-actor effect moderated by the partner's prior knowledge). Both indirect effects became smaller with increasing prior knowledge. However, while the actor-actor effect became even significantly negative at relatively high prior knowledge, the partner-actor effect only decreased to a non-significant level (compare Figures 5A, B). This pattern of results seems reasonable when considering that the actor's constructive and interactive activities together form the input that goes beyond the instructional material and that the partner can use for his or her interactive activities during collaboration (e.g., Chi and Wylie, 2014). Thus, if the effect of one's own constructive preparation activities on one's own interactive collaboration activities is initially positive, then non-significant, and finally negative with increasing own prior knowledge, it seems plausible that this also applies in a weakened form to the interactive activities of the partner.

Our results further revealed the indirect effect of the co-learners' constructive preparation activities on one's own deep comprehension outcomes via own interactive activities to not only depend on the co-learner's but also one's own prior knowledge (partner-actor effect moderated by actor prior knowledge). This is because the partner's enacted constructive preparation activities fostered one's own execution of interactive activities only when own prior knowledge was at relatively higher levels (starting from the 50^th^ percentile). This suggests that taking up and integrating the externalizations resulting from the co-learner's constructive preparation activities in the sense of own interactive activities requires at least some prior knowledge to deal with the associated costs (e.g., Kalyuga, 2009; Janssen and Kirschner, 2020). Since the co-learner's constructive preparation activities per definition contain conclusions, ideas, and perspectives not already presented in the instructional material, the benefits seem to have prevailed the costs thereby, despite an already advanced own knowledge about the instructional material.

While the partner's constructive preparation activities had a positive indirect effect on one's own deep comprehension outcomes under certain conditions of own and partner's prior knowledge as described, the direct effect was negative, which seems somewhat counterintuitive. Recall, however, that a direct effect is the influence that remains after taking into account the indirect effect: accordingly, the negative direct partner effect could be interpreted in terms of the impact of constructive activities externalized by the co-learner, which are not used as a learning resource in the course of one's own interactive activities in the service of deep comprehension. Thus, as a possible explanation, the negative direct partner effect could be understood as the impact of information that makes the learning situation more complex and increases the demands on one's own cognitive resources while not contributing to one's own learning (Kirschner et al., 2018; cf. Janssen and Kirschner, 2020).

To conclude, our results suggest that executing constructive activities during individual preparation seems to yield advantages as well as disadvantages in view of the learner's personal profit from subsequent collaboration in terms of in-depth knowledge acquisition. Whether the advantages or disadvantages prevail seems to depend on whose constructive activities we are asking about and who brings how much prior knowledge to the table. The outlined insights provide hints as to where approaches might be taken in order to optimize the design of the individual preparation and the subsequent collaboration phase so that learners could be better and more reliably supported in benefitting from their own and each other's executed constructive preparation activities in view of deep learning from subsequent collaboration. Among others, this is addressed in the next section.

### 4.2 Limitations and future directions

This work is subject to several limitations, pointing at the need for further developments and investigation in future studies. These concern (a) the instructional design choices, (b) the study variables considered, and (c) the design of the present study.

#### 4.2.1 Instructional design choices

The findings of the present study must be seen in the light of the instructional design choices applied in the study. First, the preparation tasks were provided without any further support or guidance. Numerous practicable approaches are available in this regard, which can be implemented easily and flexibly thanks to digital technologies. For example, structuring scaffolds such as prompts pointing at important information or guidelines on how to decompose a complex problem may especially support learners with low prior knowledge in performing (more) constructive activities in response to highly generative tasks (e.g., Reiser, 2004; Kalyuga, 2009). Future studies should therefore investigate whether the interaction between prior knowledge and the generativity of preparation tasks demonstrated here still holds when the latter are provided with additional support. However, it is an open issue for further research whether such support for learners with higher prior knowledge would be redundant and, thus, ineffective or even detrimental to their learning process (e.g., Kalyuga et al., 2003; Chen et al., 2017). Hence, it deserves further attention how the interplay between task generativity, prior knowledge, and supporting scaffolds might affect learners' execution of constructive preparation activities. Respective insights could inform adaptive support strategies which help to streamline generative preparation task effectiveness.

Second, within the individual preparation phases, after completing the individual externalization task, learners received each other's individual task responses (awareness induction), with these responses being presented exactly as written by the co-learner and without specific instructions on how to process the response in order to further prepare for collaboration or using them during collaboration. In addition, a relatively general instruction was intentionally used for the subsequent collaboration phase: to work together to help each other improve their understanding about the instructional material. Future work could examine how the awareness induction and collaboration phases could be optimized so that the benefits of constructive preparation activities can be enhanced and the disadvantages mitigated. For instance, data mining techniques could be applied to filter and (graphically) organize the co-learner's task responses received during awareness induction, which could facilitate their processing and comparison with one's own response (e.g., Erkens et al., 2016; Bodemer et al., 2018). This might reduce the costs imposed on oneself through the externalized constructive preparation activities of co-learners, so that their negative direct effect on one's own deep comprehension outcomes could be reduced in favor of a greater positive indirect effect via one's own interactive activities (cf. Janssen and Bodemer, 2013). In addition, this could also allow learners with little prior knowledge to use and benefit from their partner's externalized constructive preparation activities. As another example, learners could be scripted to go through their preparation products with each other step by step during collaboration, with the task of reaching explicit consensus at each step (e.g., Kollar et al., 2014; Lam and Muldner, 2017). Among other things, this could stimulate also learners with high prior knowledge to progress from their previously externalized constructive preparation activities to more (rather than less) interactive activities because they have to explicitly discuss their individually prepared thoughts and conclusions with their co-learner.

Third, considering the ubiquitous use of digital technologies in all areas of human life, the relevance and ecological validity of this work is very high. Computer use yields many advantages over analog solutions, especially in synchronous learning (e.g., Jeong and Hmelo-Silver, 2016): digital technologies facilitate the collection, distribution, presentation, and graphical organization of group awareness information such as individual task solutions or dialogue protocols (e.g., chat histories). Further, learners can be presented with virtual environments or interfaces tailored to the collaborative task at hand as well as their individual prerequisites and, thus, allow for the ergonomic and effective implementation of collaboration scripts. In addition, realizing synchronous learning via chat rooms allows many groups to interact in the same physical room without disturbing each other. Last but not least, computer techniques facilitate researcher's data collection since the results of learners' activities can be logged automatically. An interesting question for future research would be, whether the present results could be replicated in an asynchronous digital learning setting.

#### 4.2.2 Study variables

As a first issue, in contrast to the (potential) benefits, we considered the costs of collaboration not explicitly in the form of process measures. This is also indicated in the results: Regarding the effects of actor's and partner's constructive preparation activities on deep comprehension outcomes, we found not only indirect effects via the interactive activities, but also direct effects. In the literature on mediation analysis (e.g., Zhao et al., 2010), this is typically seen as a reason to search for hitherto unaccounted mediators in future studies. In our view, this applies less to the direct positive actor effect, since the latter can be interpreted reasonably as the learning effect of performing constructive activities, so that the question of mediating variables does not necessarily arise here. With regard to the negative direct partner effect, on the other hand, the search for previously omitted mediators seems more advisable. One candidate would be, for example, the mental effort invested by the learners during the collaboration (e.g., Janssen et al., 2010; Zambrano et al., 2019). Future studies should include also such or similar mediators to not only capture processes associated with the benefits (such as interactive collaboration activities) but also with the costs of collaboration more explicitly. This could add to the picture of the mechanisms underlying the advantages and disadvantages of constructive preparation activities in view of deep learning from subsequent collaboration.

Secondly, the coding of the preparation and collaboration activities according to ICAP could be further differentiated according to other available coding schemes. Though the ICAP framework provides clear criteria concerning the quality an activity must have to be coded, for example, as a constructive activity (i.e., must contain an inference), these classes are quite broad and domain general. In future studies constructive and interactive activities could, for instance, each be assessed with respect to whether they have the structure of more or less complete arguments or explanations.

Thirdly, this study considered the effects of constructive preparation activities on subsequent deep learning from collaboration exclusively through a cognitive lens. Future studies could expand the picture by also taking into account metacognitive and motivational variables as mediators and/or learning outcomes. For example, confidence judgments measured between preparation and collaboration phases (e.g., Schnaubert and Bodemer, 2019) could be examined as a possible mediator of the effect of one's own constructive preparation activities on one's own interactive collaboration activities, thus providing more insight into the possible reasons for the effect's dependence on own prior knowledge. Alternatively, or in addition, experienced curiosity could be a motivational mediator candidate here (cf. Glogger-Frey et al., 2015).

#### 4.2.3 Design

As a first issue, we excluded students of medicine, biology etc. Though this decision was made to avoid ceiling effects, it limits the generalizability of the results to learners with low prior knowledge with regard to the learning domain. At the same time, when interpreting the present results, it should be taken into account that the learning topic (i.e., circulatory system) did not play a role in the education program of the subjects studied, which may have limited their learning motivation.

Secondly, it is possible that the effects of constructive preparation activities (also) depend on prior-knowledge related dyad composition (i.e., homogeneous or heterogeneous; cf. Janssen and Kirschner, 2020). However, the analyses we conducted in response to RQ 2 did only allow for conclusions about the effects of constructive preparation activities in dependence of the actor's *or* the partner's prior knowledge. A proper investigation of this issue would require the analysis of higher-order interactions (e.g., actor constructive activities x actor prior knowledge x partner prior knowledge). To this end, future studies should employ a larger sample size and/or a priori grouping by prior knowledge to ensure that a wide range of value combinations of own and partner prior knowledge are each present in sufficiently large numbers of cases (i.e., dyads).

Thirdly, the findings concerning the deep comprehension results must be seen in the light of the delay of 1 week at which the posttest was administered. More concretely, the missing benefits of the explanation and compare-contrast as compared to the note-taking conditions might be (in part) a result of the noise caused by the delay. Thus, it is an open question for further research if potential benefits of more rather than less generative tasks would be more apparent in an immediate posttest.

## 5 Conclusion

This study contributes in several ways to our understanding of how more or less generative preparation tasks can influence learners' individual execution of constructive preparation activities and later collaborative learning. It also highlights aspects which should be taken into consideration in future investigations and in the instructional design of CSCL arrangements.

First, our findings suggest that is not so much the preparation task itself but rather what learners actually do with it that is critical to the subsequent collaboration quality and deep comprehension outcomes. This indicates that researchers should not only manipulate learning conditions involving different preparation tasks and then capture the desired collaborative learning activities and outcomes. They should also consider the activities learners actually execute in response to the tasks during individual preparation (cf. Chi and Wylie, 2014).

Second, this study highlights that data analyses should not only include deep comprehension as a collaboration outcome but also interactive activities as a mediating process in order to obtain information about whether constructive preparation activities indeed foster deep learning from rather than irrespective of subsequent collaboration. We argue that this analytical procedure should be generally applied in investigations concerned with assessing whether a certain intervention qualifies as effective means in fostering deep *collaborative* learning.

Third, considering prior knowledge as a moderator in conjunction with the distinction between actor and partner effects provided new insights into how the learner's personal benefit-cost ratio of performing interactive collaboration activities may be affected by previous constructive preparation activities. More specifically the present findings suggest that one's own and one's co-learner's constructive preparation activities (a) related differently to these personal benefits and costs, (b) both yield potential advantages and disadvantages in this regard with, and (c) prior knowledge being critical to what prevails.

Taken together, this study indicates that increasing the generativity of individual preparation tasks fosters the learner's execution of constructive preparation activities. However, it also shows that this alone is not sufficient to subsequently promote deep learning from collaboration. To this end, the results of our detailed analyses provide concrete starting points for future research that should investigate how instructional design around generative preparation tasks can be optimized for whom, so that the disadvantages of own and co-learner's constructive preparation activities are mitigated and the advantages can unfold.

## Data availability statement

The quantitative raw data including the SPSS analysis syntax and an Excel spreadsheet that we created for the analysis are available at: https://osf.io/wq83c/.

## Ethics statement

Ethical review and approval was not required for the study on human participants in accordance with the local legislation and institutional requirements. For low-risk studies like the one described in this manuscript, there is no ethics approval required in Germany. The studies were conducted in accordance with the local legislation and institutional requirements. The participants provided their written informed consent to participate in this study.

## Author contributions

SM: Writing – review & editing, Writing – original draft, Visualization, Software, Methodology, Investigation, Conceptualization. AP: Writing – review & editing, Visualization, Supervision, Software, Methodology, Conceptualization. SN: Writing – review & editing, Visualization, Supervision.
