# Supplementary material for: Generative preparation tasks in digital collaborative learning: actor and partner effects of constructive preparation activities on deep comprehension
Source: Front Psychol. 2024 Jun 19;15:1335682. doi: 10.3389/fpsyg.2024.1335682 (PMC11220279; doi:10.3389/fpsyg.2024.1335682)
Supplement: Supplementary file 1 [file Presentation_1.pdf]

Appendices

Appendix A: Individual preparation tasks applied in the externalization phases of the experiment

- Note-taking task condition: Please take notes on the […] section of the text, as you would for an exam.
  - [first]
  - [second]
  - [third]

- Compare-contrast task condition: Using the text, please compare […], that is, what are the similarities and what are the differences in terms of their components and the processes involved in each?
  - [the atria with the ventricles]
  - [the arteries, capillaries and veins with each other]
  - [the systemic circulation with the pulmonary circulation]

- Explanation task condition: Please derive as many reasons as possible from the text to answer the following question: […]
  - [Why does the heart not consist of only one atrium or only one ventricle on each side, but has both atrium and ventricle on each side?]
  - [Why is the circulatory system not composed entirely of only arteries or only capillaries or only veins, but has arteries as well as capillaries as well as veins?]
  - [Why does the circulatory system not consist of a single large circuit connecting the heart, body, and lungs, but has a systemic circulation and a pulmonary circulation?]

Appendix B: Examples of posttest questions for the assessment of text comprehension adapted from Chi et al. (2001; pp. 522-525)

Where does the blood go when it returns from the body? (shallow)

a) To the right atrium x

b) To the right ventricle

c) To the left atrium

d) To the left ventricle

Which part of the heart is the most muscular? (deep)

a) Left atrium

b) Left ventricle x

c) Right atrium

d) Right ventricle

What would be the consequence if the valve between the right atrium and the right ventricle were stuck open and wouldn’t close? (deep)

a) Too little blood flows from the veins of the body into the right side of the heart.

b) More oxygen-rich blood flows into the lungs via the pulmonary artery

c) Too much blood flows through the pulmonary artery into the lungs.

d) Blood backs up from the right side of the heart into the body's veins x

Appendix C: See Table C

Table C. Mixed regression results for the effects of preparation task type and prior knowledge (step 1) and their interaction (step 2).

Dependent variable		**Constructive preparation activities**					
Model	Null	Step 1			Step 2		
Predictor		*B*	Boot SE	BCa *CI95%*	*B*	Boot SE	BCa *CI95%*
Note-taking vs. compare-contrast (NO-CC)		6.59	.65	**[5.27, 7.91]**	6.72	.63	**[5.47, 8.02]**
Note-taking vs. explanation (NO-EX)		11.18	.67	**[9.88, 12.40]**	11.03	.61	**[9.82, 12.31]**
Compare-contrast vs. explanation (CC-EX)[a]		4.59	.76	**[3.04, 6.10]**	4.31	.80	**[2.78, 5.75]**
Actor's prior knowledge (PK_A)		.30	.05	**[.21, .39]**	.03	.05	[-.06, .13]
Partner's prior knowledge (PK_P)		.04	.04	[-.04, .13]	.08	.06	[-.03, .19]
NO-CC * PK_A					.41	.11	**[.20, .70]**
NO-CC * PK_P					.07	.11	[-.15, .26]
NO-EX * PK_A					.35	.08	**[.18, .50]**
NO-EX * PK_P					-.08	.09	[-.24, .11]
CC-EX * PK_A[a]					-.06	.12	[-.32, .14]
CC-EX * PK_P[a]					-.14	.11	[-.36, .10]
ICC[b]	.63***	.23			.26		
-2Log Likelihood	763.12	666.40			653.22		
Model fit increase[c]		$\chi^2(4)^d = 96.72^{***}$			$\chi^2(4)^d = 13.18^{*}$		
Pseudo-R^2		.63			.65		

Dependent variable		**Constructive collaboration activities**					
Model	Null	Step 1			Step 2		
Predictor		*B*	Boot SE	BCa *CI95%*	*B*	Boot SE	BCa *CI95%*
Note-taking vs. compare-contrast (NO-CC)		-.38	.38	[-1.09, .36]	-.37	.40	[-1.15, .38]
Note-taking vs. explanation (NO-EX)		-.77	.31	**[-1.36, -.15]**	-.75	.32	**[-1.41, -.12]**
Compare-contrast vs. explanation (CC-EX)[a]		-.39	.32	[-1.01, .21]	-.38	.35	[-1.08, .30]
Actor's prior knowledge (PK_A)		.05	.02	**[.01, .09]**	.08	.04	**[.01, .15]**
Partner's prior knowledge (PK_P)		-.05	.02	**[-.09, -.02]**	-.06	.04	[-.15, .03]
NO-CC * PK_A					-.03	.07	[-.16, .12]
NO-CC * PK_P					.01	.07	[-.13, .14]
NO-EX * PK_A					-.05	.05	[-.15, .06]
NO-EX * PK_P					.02	.05	[-.08, .11]
CC-EX * PK_A[a]					-.02	.06	[-.14, .08]
CC-EX * PK_P[a]					.01	.06	[-.11, .13]
ICC[b]	.00	.07			.07		
-2Log Likelihood	485.91	471.45			470.51		
Model fit increase[c]		$\chi^2(4)^d = 14.46^{**}$			$\chi^2(4)^d = .94$		
Pseudo-R^2		.08			.05		

Table C continued.

Dependent variable		**Interactive collaboration activities**					
Model	Null	Step 1			Step 2		
Predictor		*B*	Boot SE	BCa *CI95%*	*B*	Boot SE	BCa *CI95%*
Note-taking vs. compare-contrast (NO-CC)		-.05	.58	[-1.27, 1.10]	.11	.62	[-1.21, 1.36]
Note-taking vs. explanation (NO-EX)		.80	.64	[-.56, 2.20]	.92	.65	[-.46, 2.29]
Compare-contrast vs. explanation (CC-EX)[a]		.85	.63	[-.45, 2.16]	.81	.64	[-.44, 2.06]
Actor's prior knowledge (PK_A)		.11	.04	**[.04, .19]**	.12	.09	[-.09, .25]
Partner's prior knowledge (PK_P)		.04	.04	[-.03, .12]	.15	.09	[-.01, .36]
NO-CC * PK_A					.08	.12	[-.15, .34]
NO-CC * PK_P					-.10	.12	[-.32, .14]
NO-EX * PK_A					-.05	.10	[-.24, .23]
NO-EX * PK_P					-.16	.10	[-.36, .01]
CC-EX * PK_A[a]					-.13	.09	[-.31, 0.10]
CC-EX * PK_P[a]					-.06	.09	[-.25, .07]
ICC[b]	$.75^{***}$	$.76^{***}$			$.76^{***}$		
-2Log Likelihood	644.29	637.14			633.05		
Model fit increase[c]		$\chi^2(4)^d = 7.15$			$\chi^2(4)^d = 4.09$		
Pseudo-R^2		.01			.00		

Dependent variable		**Deep comprehension posttest achievement**					
Model	Null	Step 1			Step 2		
Predictor		*B*	Boot SE	BCa *CI95%*	*B*	Boot SE	BCa *CI95%*
Note-taking vs. compare-contrast (NO-CC)		-7.47	3.04	**[-13.55, -1.29]**	-7.41	3.18	**[-13.77, -.89]**
Note-taking vs. explanation (NO-EX)		-1.19	3.01	[-7.38, 4.76]	-1.08	3.17	[-7.33, 5.16]
Compare-contrast vs. explanation (CC-EX)[a]		6.28	2.91	**[.54, 11.76]**	6.33	3.02	**[.10, 12.25]**
Actor's prior knowledge (PK_A)		1.26	.19	**[.87, 1.60]**	.95	.45	**[.02, 1.57]**
Partner's prior knowledge (PK_P)		-.06	.20	[-.46, .37]	.38	.50	[-.45, 1.64]
NO-CC * PK_A					.25	.62	[-.93, 1.88]
NO-CC * PK_P					-.40	.63	[-1.60, .52]
NO-EX * PK_A					.46	.53	[-.55, 1.70]
NO-EX * PK_P					-.67	.58	[-1.88, .32]
CC-EX * PK_A[a]					.22	.52	[-.82, 1.04]
CC-EX * PK_P[a]					-.28	.50	[-1.28, .91]
ICC[b]	.08	.10			.13		
-2Log Likelihood	1050.31	1006.01			1002.90		
Model fit increase[c]		$\chi^2(4)^d = 44.30^{***}$			$\chi^2(4)^d = 3.10$		
Pseudo-R^2		.28			.27		

Note. All continuous predictors were centered prior to analyses. Unstandardized regression coefficients are reported. Accelerated and bias-corrected bootstrap confidence intervals indicating significant regression weights are written in bold.

[a] Each of the presented models were computed twice: one time with NO-CC and NO-EX and one time with NO-CC and CC-EX. [b] Intraclass-correlation [c] Model fit increase as determined by -2 Log Likelihood differences. [d] In comparison to previous model.

* $p < .05$, $^{**}p < .01$, *** $p < .001$.

Appendix D: See Table D

Table D. Mixed regression results of the moderated mediation analyses

Dependent variable (path)	**Constructive collaboration activities (a-path)**						
Model	Null	Step 1			Step 2		
Predictor		*B*	Boot SE	BCa *CI95%*	*B*	Boot SE	BCa *CI95%*
Actor‘s prior knowledge (PK_A)		.05	.02	**[.01, .09]**	.05	.02	**[.01, .10]**
Partner’s prior knowledge (PK_P)		-.04	.02	**[-.09, -.00]**	-.04	.03	[-.10, .00]
Actor’s constructive preparation activities (CP_A)		.01	.04	[-.07, .09]	.02	.04	[-.05, .09]
Partner’s constructive preparation activities (CP_P)		-.03	.04	[-.11, .06]	-.04	.05	[-.14, .07]
$CP_A * PK_A$					-.00	.01	[-.01, .00]
$CP_P * PK_P$					-.00	.01	[-.01, .02]
$CP_A * PK_P$					.01	.01	[-.01, .01]
$CP_P * PK_A$					.00	.01	[-.01, .02]
ICC[a]	.00	.08			.09		
-2Log Likelihood	485.91	474.60			472.29		
Model fit increase[b]		$\chi^2(4)^c = 11.31^*$			$\chi^2(4)^c = 2.31$		
Pseudo-R^2		.05			.04		
Dependent variable (path)	**Interactive collaboration activities (a-path)**						
Model	Null	Step 1			Step 2		
Predictor		*B*	Boot SE	BCa *CI95%*	*B*	Boot SE	BCa *CI95%*
Actor‘s prior knowledge (PK_A)		.10	.04	[-.00, .18]	.13	.05	**[.03, .20]**
Partner’s prior knowledge (PK_P)		-.00	.05	[-.09, .09]	.02	.04	[-.07, .12]
Actor’s constructive preparation activities (CP_A)		.05	.05	[-.05, .17]	.07	.06	[-.03, .20]
Partner’s constructive preparation activities (CP_P)		.16	.06	**[.03, .27]**	.24	.07	**[.08, .36]**
$CP_A * PK_A$					-.03	.01	**[-.04, -.01]**
$CP_P * PK_P$					-.03	.01	**[-.04, -.01]**
$CP_A * PK_P$					.01	.01	[-.01, .03]
$CP_P * PK_A$					.03	.01	**[.01, .05]**
ICC[a]	$.75^{***}$	$.74^{***}$			$.71^{***}$		
-2Log Likelihood	644.29	630.40			615.97		
Model fit increase[b]		$\chi^2(4)^c = 13.89^{**}$			$\chi^2(4)^c = 14.43^{**}$		
Pseudo-R^2		.09			.20		

Table D continued.

Dependent variable (path)	Deep comprehension posttest achievement (b-path)						
Model	Null	Step 1			Step 2		
Predictor		*B*	Boot SE	BCa *CI95%*	*B*	Boot SE	BCa *CI95%*
Actor's prior knowledge (PK_A)		.98	.21	**[.55, 1.38]**	1.09	.24	**[.60, 1.55]**
Partner's prior knowledge (PK_P)		.19	.21	[-.20, .59]	.25	.24	[-.20, .70]
Actor's constructive preparation activities (CP_A)		.63	.29	**[.05, 1.20]**	.45	.37	[-.28, 1.08]
Partner's constructive preparation activities (CP_P)		-.69	.29	**[-1.22, -.15]**	-.42	.37	[-1.18, .42]
Actor's interactive collaboration activities (IC_A)		1.75	.48	**[.82, 2.75]**	1.63	.60	**[.52, 2.71]**
Partner's interactive collaboration activities (IC_P)		-.86	.49	[-1.93, .04]	-.78	.58	[-2.00, 0.44]
CP_A * PK_A					-.05	.05	[-.16, .02]
CP_P * PK_P					.00	.04	[-.08, .11]
CP_A * PK_P					-.01	.06	[-.12, .08]
CA_P * PK_A					.12	.07	[-.01, .30]
IC_A * PK_A					-.05	.08	[-.20, .08]
IC_P * PK_P					-.10	.08	[-.26, .07]
IC_A * PK_P					.13	.08	[-.00, .27]
IC_P * PK_A					-.01	.08	[-.20, .20]
ICC[a]	.08	.19			.18		
-2Log Likelihood	1050.31	992.90			981.70		
Model fit increase[b]		$\chi^2(6)^c = 57.41^{***}$			$\chi^2(8)^c = 11.20$		
Pseudo-R^2		.33			.35		

Note. Since constructive collaboration activities disqualified for being a mediator during a-path analyses, they are not included within b-path analyses. All continuous predictors were centered prior to analyses. Unstandardized regression coefficients are reported. Accelerated and bias-corrected bootstrap confidence intervals indicating significant regression weights are written in bold.

[a] Intraclass-correlation [b] Model fit increase as determined by -2 Log Likelihood differences. [c] In comparison to previous model.

* $p < .05$, $^{**}p < .01$, *** $p < .001$.
